# Distinct 5-methylcytosine profiles in poly(A) RNA from mouse embryonic stem cells and brain

**DOI:** 10.1186/s13059-016-1139-1

**Published:** 2017-01-05

**Authors:** Thomas Amort, Dietmar Rieder, Alexandra Wille, Daria Khokhlova-Cubberley, Christian Riml, Lukas Trixl, Xi-Yu Jia, Ronald Micura, Alexandra Lusser

**Affiliations:** 1 Division of Molecular Biology, Biocenter, Medical University of Innsbruck, 6020 Innsbruck, Austria; 2 Division of Bioinformatics, Biocenter, Medical University of Innsbruck, 6020 Innsbruck, Austria; 3 Zymo Research corp, Irvine, CA USA; 4 Department of Organic Chemistry and Center for Molecular Biosciences (CMBI), Leopold-Franzens University, 6020 Innsbruck, Austria

**Keywords:** RNA methylation, 5-Methylcytosine, m5C, Epitranscriptome, Embryonic stem cells, Mouse brain, m6A, RNA binding proteins, Bisulfite sequencing, meRIP

## Abstract

**Background:**

Recent work has identified and mapped a range of posttranscriptional modifications in mRNA, including methylation of the N6 and N1 positions in adenine, pseudouridylation, and methylation of carbon 5 in cytosine (m5C). However, knowledge about the prevalence and transcriptome-wide distribution of m5C is still extremely limited; thus, studies in different cell types, tissues, and organisms are needed to gain insight into possible functions of this modification and implications for other regulatory processes.

**Results:**

We have carried out an unbiased global analysis of m5C in total and nuclear poly(A) RNA of mouse embryonic stem cells and murine brain. We show that there are intriguing differences in these samples and cell compartments with respect to the degree of methylation, functional classification of methylated transcripts, and position bias within the transcript. Specifically, we observe a pronounced accumulation of m5C sites in the vicinity of the translational start codon, depletion in coding sequences, and mixed patterns of enrichment in the 3′ UTR. Degree and pattern of methylation distinguish transcripts modified in both embryonic stem cells and brain from those methylated in either one of the samples. We also analyze potential correlations between m5C and micro RNA target sites, binding sites of RNA binding proteins, and *N*6-methyladenosine.

**Conclusion:**

Our study presents the first comprehensive picture of cytosine methylation in the epitranscriptome of pluripotent and differentiated stages in the mouse. These data provide an invaluable resource for future studies of function and biological significance of m5C in mRNA in mammals.

**Electronic supplementary material:**

The online version of this article (doi:10.1186/s13059-016-1139-1) contains supplementary material, which is available to authorized users.

## Background

Posttranscriptional modification of RNA has been known for longer than 70 years. To date, more than 140 modifications that map to all bases as well as the ribose moiety have been discovered in the abundant non-coding RNAs of the cell, in particular in transfer and ribosomal RNAs (tRNAs and rRNAs) [1]. By contrast, much less is known about base modifications in poly(A) RNAs [2–4]. Only recently, with the advent of techniques enabling transcriptome-wide position-specific determination of base modifications, specifically methylation, has this area attracted a surge of attention. It has become clear that posttranscriptional RNA modification may impose an additional level on transcript regulation. Similar to what is known from chromatin, where modifications of the DNA and histones have been recognized as important regulators of genomic information and are therefore part of the “epigenome,” the ongoing discovery of distinct RNA modifications has prompted the coining of the terms “RNA epigenetics” [5] and “epitranscriptomics” [6, 7]. To date, the best studied modification of poly(A) RNA is *N*6-methyladenosine (m6A) and, in analogy to the epigenetic code, “writers,” “erasers,” and “readers” of this modification have been identified [8–12]. Recent work has shown that m6A affects transcript splicing, stability, translation, and nuclear export [13–18], and inactivation of the responsible methyltransferase complex METTL3/METTL14/WTAP severely impairs embryonic stem cell differentiation and results in early embryonic lethality [15, 19]. Pseudouridine and *N*1-methyladenosine (m1A) are further modifications that have recently been discovered on a transcriptome-wide level in mammalian RNA [20–23], yet their functional impact has not been studied yet.

In addition to these modifications, it has been known since the 1970s that the C5 atom of cytosine can be a target of methylation in poly(A) RNA in HeLa and hamster cells [24, 25]. By contrast, other early studies failed to detect m5C in mRNA [26, 27]. Due to the lack of suitable methodology, research on m5C all but ceased for several decades. Several enzymes belonging to the RNCMT (RNA (cytosine-5) methyltransferase) family of proteins have been shown to act as cytosine methyltransferases for tRNAs and rRNAs using a catalytic mechanism that involves transient formation of a covalent enzyme-cytosine adduct [3, 28]. By exploiting this property, two recent studies reported the transcriptome-wide mapping of m5C sites generated by the methyltransferases NSUN2 and DNMT2, respectively, in the mouse and in human cell lines [29, 30]. It was shown that both enzymes preferentially target tRNAs, and that NSUN2 also modifies the highly abundant vault RNAs [30]. The adaptation of the bisulfite sequencing technique that is widely used to study DNA methylation for application with RNA [31] enabled the unbiased mapping of m5C sites in poly(A) RNA in a transcriptome-wide manner. To date, only two studies have used this technique to investigate global m5C in human HeLa cells [32] and in archeal mRNA, respectively [33]. Both studies revealed widespread occurrence of m5C in poly(A) RNA. We have previously shown that the long non-coding RNAs XIST and HOTAIR are methylated in vivo and that the methylation interferes with binding of XIST to Polycomb repressive complex 2 (PRC2) in vitro [34].

Thus, in this work, we aimed at obtaining a deeper understanding of m5C methylation in poly(A) RNA in the mouse. To this end, we mapped m5C globally using RNA bisulfite sequencing (RNA BS-seq) in embryonic stem cells (ESCs) and the brain in total and nuclear poly(A) RNA and compared its prevalence and distribution in both cell/tissue types and cellular compartments. In addition, we examined potential links to micro RNA (miRNA) and protein binding sites and m6A patterns. Collectively, these data constitute a comprehensive picture of cytosine methylation in poly(A) RNA of different cell types/tissues in the mouse and provide the basis for future studies of its function and biological significance in mammals.

## Results

### Bisulfite sequencing of nuclear and total poly(A) RNA in embryonic stem cells and mouse brain

#### Bisulfite treatment, m5C calling, and controls

To gain an overview of transcriptome-wide cytosine methylation, we performed bisulfite sequencing (BS-seq) of RNA derived from mouse ESCs and from the adult mouse brain. We prepared poly(A)-enriched RNA from three biological replicates of both samples and performed three cycles of bisulfite treatment followed by deep sequencing using the Illumina HiSeq platform. In addition, we performed the same experiments with poly(A) RNA isolated from purified nuclei of ESC and brain. To control for efficient bisulfite-mediated C → U conversion, the samples were supplemented with in vitro transcribed and folded RNA templates corresponding to nucleotides (nt) 914–1465 of *Escherichia coli* 16S rRNA (ESC and brain) as well as a transcript corresponding to ~5700 nt of the pET-15b vector sequence (ESC). On average, we obtained ~58 million unambiguously mapped reads for each of three brain replicates and ~40 million unambiguously mapped reads for each ESC replicate (Additional file 1). For high-confidence mapping and m5C calling, we developed a specialized bioinformatics tool package [35]. Using this pipeline, the vast majority of reads could be aligned to the mouse reference genome (GRCm38/mm10) with 0–1 mismatches (Additional file 2: Figure S1). Analysis of the spike-in controls revealed C → U conversion rates >99% (Additional file 3). For m5C calling, we considered only positions that were covered by >10 reads and showed a non-conversion rate of >20% and a methylation state false discovery rate (FDR) <0.01 (calculated using spike-in control conversion rates as described in [35]). In addition, candidate m5Cs had to be present in all three replicates. Using these parameters, we detected zero m5Cs in the 16S rRNA yet one position in the pET vector spike-in control (Additional file 2: Figures S2 and S3). Since efficient bisulfite treatment requires that the cytosines are single stranded, we introduced an additional filtering step to the m5C dataset to eliminate potential false positive candidates arising from putative secondary structure formation. To this end, we retrieved all full-length transcripts containing an m5C candidate from the RefSeq database (GRCm38.p3) and subjected them to secondary structure prediction using the RNAfold algorithm (see Methods for details). We then discarded all m5Cs that were predicted to be in a base-paired state. These highly stringent filtering parameters also successfully eliminated the single false positive in the spike-in controls (Additional file 2: Figure S3).

#### Total poly(A) RNA

**Fig. 1:**
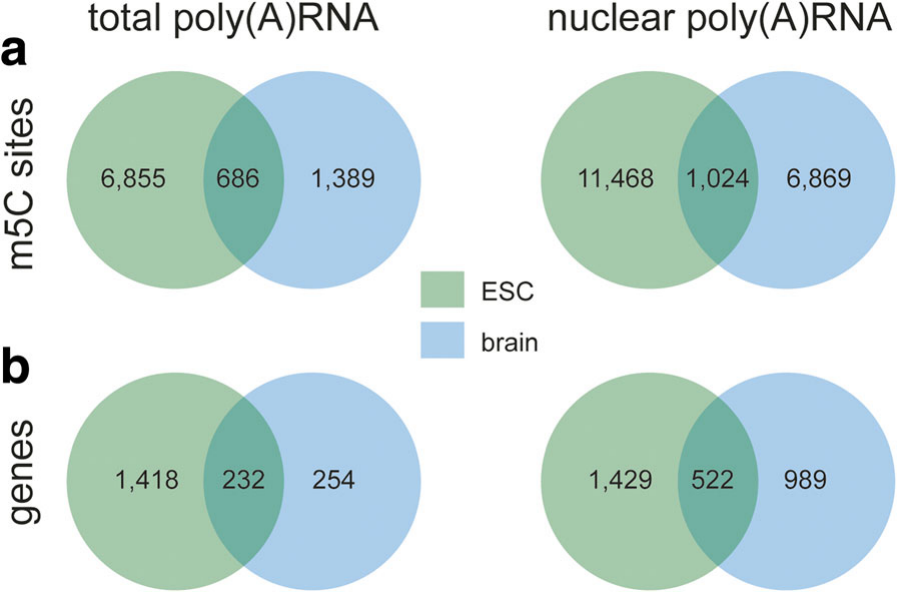
BS-seq of total and nuclear poly(A) RNA samples from ESCs and brain reveals shared and sample-specific methylation sites. **a** Venn diagrams of methylation sites identified in total poly(A) RNA (*left*) or nuclear poly(A) RNA (*right*) from mouse ESC and brain. **b** Venn diagrams of number of genes to which identified m5Cs were mapped

Applying these parameters to our total poly(A) RNA, we discovered 7541 m5C candidate sites in ESCs and 2075 m5C candidates in the brain (Fig. 1a, Additional files 4 and 5). Mapping of the methylated positions to the reference genome revealed their location in 1650 (ESC) and 486 (brain) annotated genes, respectively (Fig. 1b), which corresponds to 11% (ESC) and 3% (brain) of all genes for which we detected expression with more than 10 reads (mean normalized read count; Additional file 6). Comparing the data from ESCs with those from brain also revealed that most of the identified sites were specific to ESC (90%) and brain (67%), respectively (Fig. 1a), meaning that they appeared in all three replicates of one sample but in fewer than three replicates of the other. Interestingly, the data also suggest that the number of methylated sites per gene is higher in transcripts found specifically methylated in either ESC or brain (ESC: 4.8 sites/gene; brain: 5.5 sites/gene) compared to transcripts methylated in both samples (3 sites/gene). However, it is important to note that due to the short sequencing read lengths, it is not possible to determine the methylation state of individual full-length mRNA molecules, and thus these numbers are merely rough estimates. Taken together, the results imply that (1) the overall frequency of m5C occurrence is higher in ESC than in brain samples, (2) the diversity of methylated transcripts is higher in ESCs compared to brain, and (3) transcripts methylated in one sample but not the other tend to have higher numbers of m5Cs than transcripts methylated in both samples.

#### Nuclear poly(A) RNA

As the poly(A) RNA fraction of total RNA contains both cytoplasmic and preprocessed transcripts as well as mature transcripts located in the nucleus, we were interested to learn whether there is a difference between m5C distribution in the total RNA-derived fraction and nuclear RNA. Therefore, we prepared poly(A) RNA from isolated nuclei of ESCs and the brain for bisulfite treatment and sequencing applying identical quality control and analysis parameters as before (Additional file 1). We found almost twice as many m5C sites (12,492) in nuclear RNA of ESCs and almost four times more m5C sites (7893) in brain nuclear RNA compared to the corresponding total poly(A) RNA samples (Fig. 1a, Additional files 7 and 8). These sites mapped to 1951 genes in ESCs and 1511 genes in the brain (Fig. 1b). Similar to the findings for total poly(A) RNA, the majority of m5C candidate sites were specific to the sample type (92% in ESCs, 87% in brain). Also, the number of m5C sites per gene was higher in transcripts methylated in one sample compared to those methylated in both samples. Unlike in the total poly(A) RNA samples, however, the frequency of methylation in the sample-specific methylated transcripts was slightly lower in brain (6.9 sites/gene) than in ESCs (8 sites/gene), while the opposite trend was apparent in total poly(A) RNA. We also detected several non-coding RNAs in our samples (Additional files 4, 5, 7, and 8). For example, the highly expressed long non-coding RNA (lncRNA) Malat1 was found to contain methylated cytosines in its 5′ region in both ESC and brain (Additional files 4 and 5). However, overall the number of detected ncRNAs was small in both total and nuclear poly(A) RNA.

Taken together, these results show that there are considerable differences in m5C prevalence and distribution between ESCs and adult brain. In particular, ESCs have an overall higher degree of methylation in both total and nuclear poly(A) RNA, and these m5Cs are distributed across a wider variety of transcripts than in the brain. Furthermore, poly(A) RNA derived from nuclear RNA exhibits substantially more methylated Cs in both samples, translating into higher m5C per transcript rates than in total poly(A) RNA.

### Validation of methylation targets

As pointed out above, bisulfite-mediated deamination of cytosine is inhibited if the target cytosine is part of an RNA or DNA double strand. Although we have already applied stringent filtering to our dataset with respect to the potential of secondary structure formation, we further tested our method with strongly folded RNA oligonucleotides. To this end, we synthesized the following three RNA oligonucleotides forming highly stable hairpin structures: RNA I containing a six-nucleotide-long C:G stem and a UUCG tetraloop, RNA II corresponding to a recently published quadruplex structure [36], and RNA III corresponding to the repeat 8 region of human XIST RNA [34, 37] (Additional file 2: Figure S4). These oligos were subjected to our bisulfite treatment protocol and subsequently analyzed by mass spectrometry. The results clearly show complete conversion of all Cs to Us even in the extended C:G stem structure of RNA I (Additional file 2: Figure S4), implying that potential secondary structures in the RNA source material can be overcome by this method.

**Fig. 2:**
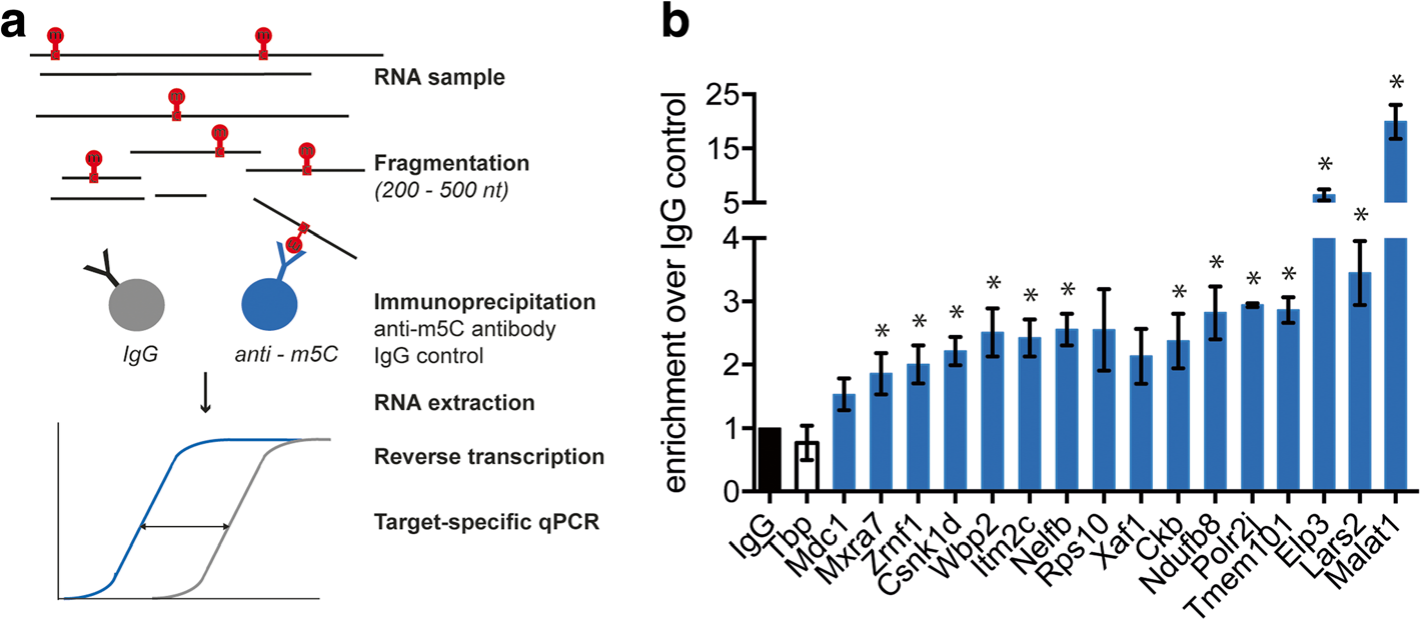
Verification of candidate methylated transcripts by meRIP. **a** Graphical depiction of the meRIP approach. RNA was extracted from cells, chemically fragmented, incubated with an anti-5-methylcytosine antibody or IgG, and antigen-antibody complexes were captured with protein A beads. Specific candidate RNAs (*blue bars* in **b**) were analyzed by qPCR of immunoprecipitated material, and enrichment relative to the IgG control (*black bar* in **b**) was calculated. **b** MeRIP shows significant enrichment of 13 out of 16 candidate transcripts. The *Tbp* transcript (*white bar*) served as a negative control, since it was not detected in our m5C dataset. Data are shown as mean ± standard error of the mean (*SEM*) of three independent experiments. Statistical significance was determined by unpaired *t* test, significance threshold *p* < 0.05 (*)

In order to validate our results from the BS-seq analysis by yet an alternative method, we chose several candidate transcripts to confirm their methylated state by methyl-RNA immunoprecipitation (meRIP) using an antibody against m5C (Fig. 2a). Using immuno-northern blot with in vitro generated control transcripts in which 0%, 50%, or 100% of all Cs were replaced by m5Cs, we first showed that the anti-m5C antibody specifically recognizes m5C-containing but not unmethylated transcripts (Additional file 2: Figure S5). Out of the 16 candidate transcripts that were analyzed, meRIP revealed significant enrichment over the IgG control reactions of 13 candidates. The TATA binding protein (*Tbp*) transcript that was not called as a methylation target in our analysis served as a negative control and showed no enrichment (Fig. 2b).

Taken together, using two alternative methods (mass spectrometry and meRIP) to validate our bisulfite treatment protocol and results, and taking into account the high deamination rates of the unmethylated spike-in controls and the stringent m5C calling parameters, we are confident that our m5C data represent a reliable picture of the methylcytosine epitranscriptome in ESCs and the mouse brain.

### Differential methylation patterns in ESC and brain are typically not caused by differential expression

**Fig. 3:**
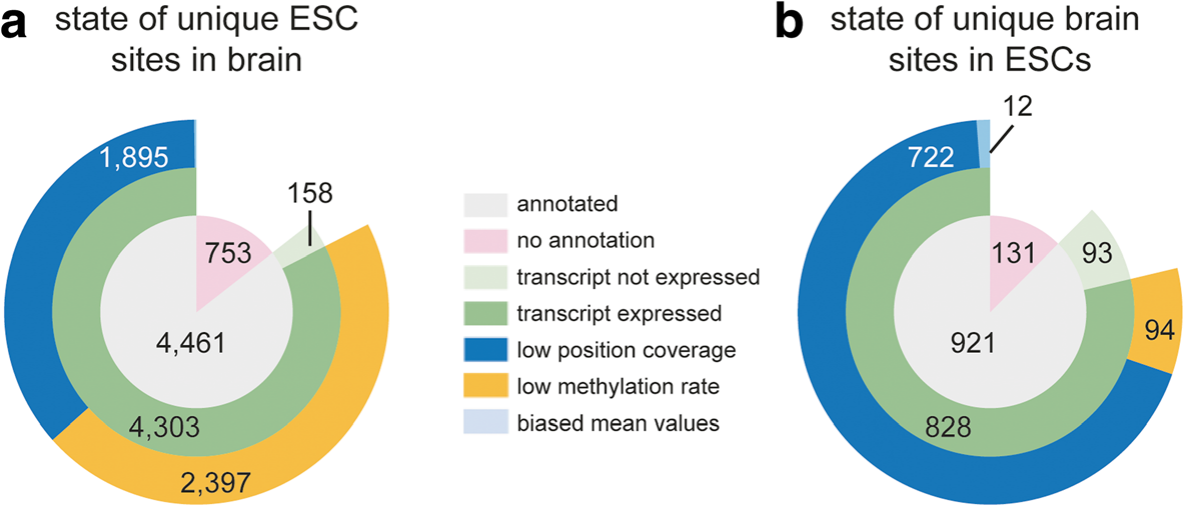
The majority of uniquely methylated cytosines in ESC total poly(A) RNA are due to differential methylation rather than differential expression between ESC and brain. **a** The expression levels and methylation rates of m5Cs identified as unique to ESCs were analyzed in the brain samples. **b** The expression levels and methylation rates of m5Cs identified as unique to brain were analyzed in the ESC samples. Multi-level pie charts display the numbers of sites on annotated and non-annotated transcripts in the *innermost ring*, the numbers of sites on transcripts with a mean normalized count of more (*dark green*) or fewer (*light green*) than 10 reads in the *middle ring*, and the numbers of sites with sequence coverage <10 reads (*blue*) or sequence coverage >10 reads but methylation rate lower than 0.2 (*yellow*) in the *outer ring*. Positions in which the mean values for coverage and non-conversion were skewed towards methylation by an individual replicate were classified as *biased mean*

To examine sample-dependent differences observed in the methylation patterns of ESC and brain, we assigned the identified methylated sites to three groups: *unique* methylation sites in ESCs and brain, respectively (these two groups comprise sites that were found methylated in three replicates of one but in none of the other sample), and *common* methylated sites (those found in three replicates of one and in at least one replicate of the other sample). We then determined if the sites present in the *unique* group were not present in the other sample because they were on transcripts not expressed in the other sample or the site was not covered by >10 reads, or if they were not methylated above the threshold of 0.2 even though the sequencing coverage of the site was sufficient in the other sample. We found 4461 uniquely methylated sites on annotated transcripts in total RNA from ESCs. Only 3% of these transcripts were expressed with a mean normalized count of <10 reads in the brain, indicating that the remaining majority of these transcripts were indeed expressed in the brain. Interestingly, 57% of the sites methylated in ESCs on these transcripts were not methylated in the brain, although the specific sites were covered by >10 reads, while 44% of the sites were not covered by enough reads to make the cut-off for calling (Fig. 3a). Thus, we conclude that the majority of uniquely methylated sites on annotated transcripts in ESCs are due to differential methylation rather than differential or lacking expression between ESCs and brain.

When taking a closer look at the *unique* group of methylations from brain total poly(A) RNA, we observed a different picture (Fig. 3b). We found 921 unique sites on annotated transcripts. However, a larger fraction (8.8%) than in ESCs resided on transcripts not expressed in ESCs. Also, the vast majority of sites on the expressed transcripts (87%) were not covered by enough reads in ESCs to match the m5C calling criteria, indicating low overall expression of the respective transcripts in ESCs. Eleven percent of the uniquely methylated sites on annotated transcripts from the brain showed clear differential methylation, as they were sufficiently covered by sequencing but did not reach the limit of 20% methylation in ESCs (Fig. 3b). Collectively, these results suggest that cytosine methylation in mRNAs can occur in a highly cell/tissue type-specific manner that is independent of transcript expression levels and that this appears to be an ESC-specific feature.

We also performed the same analyses for the analogous samples from nuclear poly(A) RNA. However, in that case the fraction of sites that did not reach sufficient read coverage in the opposite sample was much higher (especially for the brain samples), suggesting that low expression was the major reason for the occurrence of uniquely methylated cytosine positions (Additional file 2: Figure S6).

### Cytosine methylated transcripts are involved in general and cell type-specific functional pathways

**Fig. 4:**
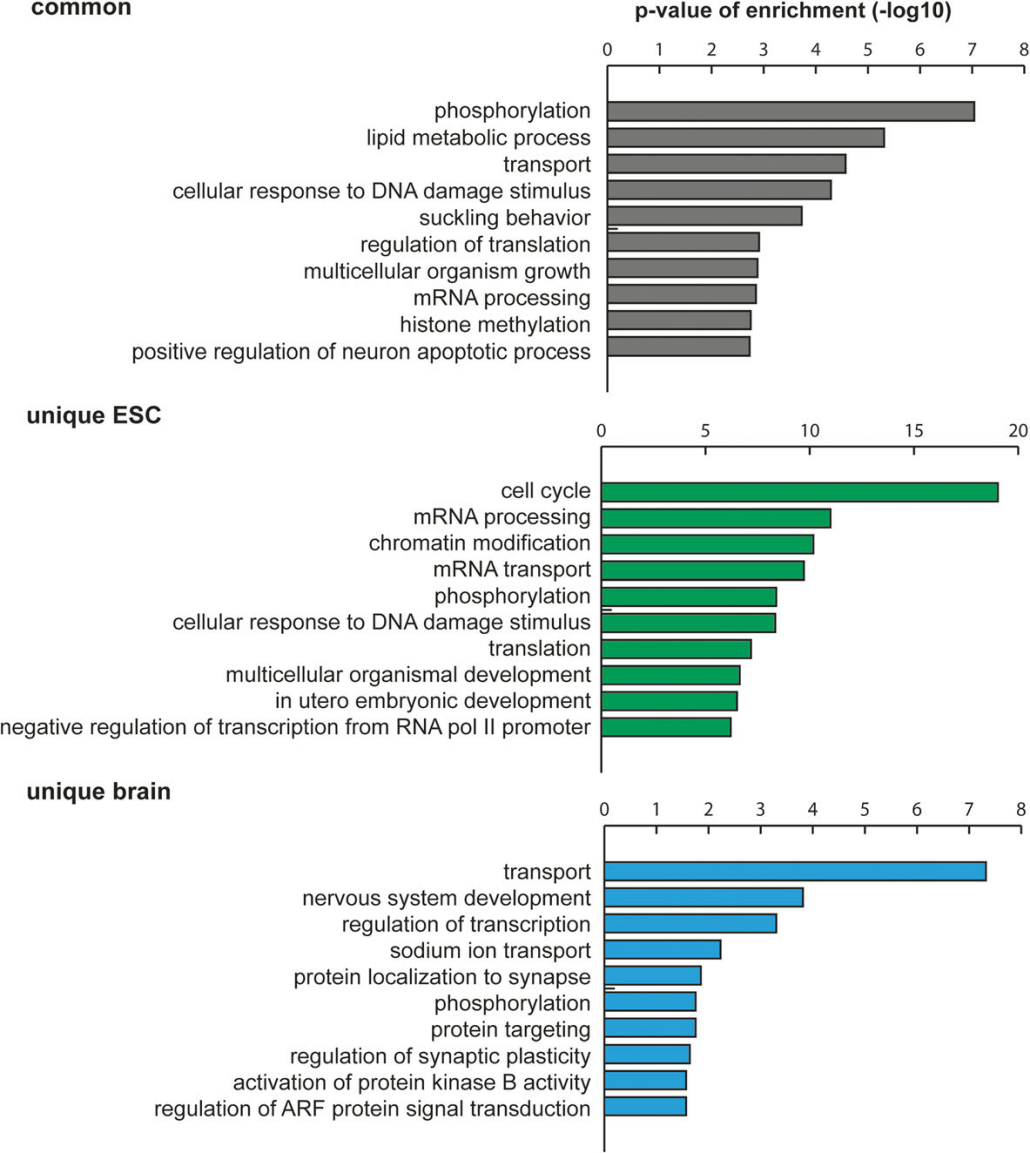
GO term enrichment analysis reveals distinct predominance of different gene categories in transcripts methylated in both ESCs and brain (*common*) versus transcripts methylated uniquely in one of the samples (*unique*). GO terms were analyzed with DAVID and further clustered using REVIGO. The ten most significantly enriched categories are shown

To determine if cytosine methylation is linked to specific functional roles in the cell, we performed Gene Ontology (GO) term enrichment analyses of target mRNAs identified in ESCs and brain. For transcripts methylated uniquely in ESCs, we found highly significant (*p* < 0.01) enrichment of categories corresponding to cell cycle, RNA processing and transport, chromatin modification, and development-related processes, while unique brain targets showed strong overrepresentation of GO terms linked to transport, nervous system development, synapse function, and protein targeting. Lipid metabolism, phosphorylation, and transport dominated the GO term analysis of transcripts that were found to be methylated in both ESCs and the brain (Fig. 4). These results indicate that cytosine methylation affects transcripts that are important for general cell metabolism as well as for processes that reflect the specific functions of the respective cell type/tissue.

### Methylated cytosines show common and distinct distribution features in ESCs and in the brain

#### Total poly(A) RNA

**Fig. 5:**
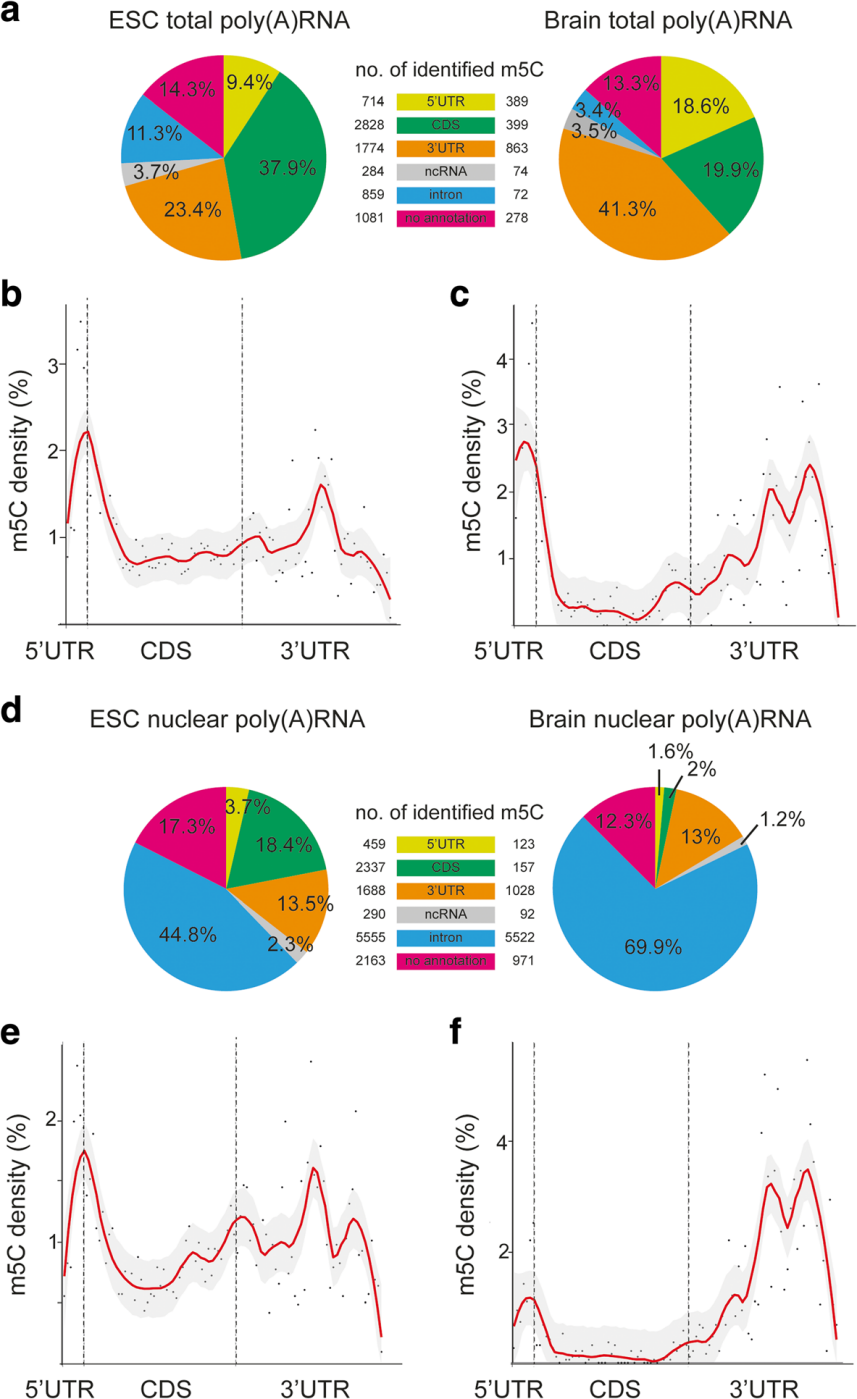
Methylated cytosines are preferentially located around the translational start codon of mRNAs. **a** The percentages of m5Cs detected in ESC (*left*) or brain (*right*) total poly(A) RNA mapping to the indicated transcript classes are shown. **b** Meta-gene profiles of all m5C locations detected in total poly(A) RNA of ESCs along the rescaled segments 5′ UTR, coding sequence (*CDS*), and 3′ UTR of a normalized mRNA are shown and indicate a peak of m5C at the translational start codon. *Red line* represents the loess smoothed conditional mean and *gray areas* the 0.95 confidence interval. *Dashed lines* separate the different mRNA segments at the translational start and stop codons. **c** Same as in **b** for brain total poly(A) RNA. **d** Pie chart of the percentages of m5Cs detected in the indicated transcript classes in ESC (*left*) or brain (*right*) nuclear poly(A) RNA. **e**, **f** Meta-gene analysis as in **b** reveals accumulation of m5C sites around the start codon in ESC (**e**) and brain (**f**) nuclear poly(A) RNA as well as in the 3′ UTR of brain nuclear RNA transcripts (**f**)

To gain a better understanding of the distribution of m5C sites in the mouse transcriptome, we examined the location of all m5Cs with respect to underlying transcript features. The majority of m5C sites were detected in the three segments of mRNA, 5′ UTR, coding sequence (CDS), and 3′ UTR, in both ESC and brain total poly(A) RNA, while about 26% (ESC) and 17% (brain) mapped to intronic and non-annotated sequences (Fig. 5a). Interestingly, there was a difference between ESC and brain, since in ESC total poly(A) RNA most methylated cytosines were detected in the coding sequence of mRNAs, while in the brain most sites were present in the 3′ UTRs (Fig. 5a). Closer inspection of the annotated mRNAs revealed significant enrichment of m5C sites in the 5′ UTR and significant depletion in the CDS in brain and ESC mRNAs (Fisher exact test; Table 1). Unexpectedly, weak depletion (odds ratio: 0.94, *p* = 0.03) was detected in the 3′ UTR of total poly(A) RNA from ESCs, but not from brain. By contrast, looking only at methylation sites shared by both samples, we found significant enrichment in the 3′ UTR, while those found in ESCs only were depleted and those found uniquely in the brain were also enriched in the 3′ UTR (Additional file 2: Figure S7).

**Table 1:** Distribution of methylated Cs in transcripts of total and nuclear poly(A) RNA of ESCs and brain

	Fisher exact test			No. of m5Cs tested
	*p* value*	Odds ratio	95% confidence interval	
Total poly(A) RNA				
ESC				
5′ UTR	1.84E-37	1.74	1.60–1.88	714
CDS	2.22E-08	0.86	0.81–0.90	2828
3′ UTR	0.033	0.94	0.89–0.99	1775
AUG (+/– 25 nt)	2.87E-29	2.38	2.07–2.72	225
Brain				
5′ UTR	1.02E-81	3.51	3.13–3.94	389
CDS	3.11E-106	0.31	0.28–0.35	399
3′ UTR	6.02E-19	1.55	1.41–1.71	863
AUG (+/– 25 nt)	6.93E-27	3.84	3.09–4.71	98
Nuclear poly(A) RNA				
ESC				
5′ UTR	2.49E-09	1.36	1.23–1.50	459
CDS	3.53E-17	0.78	0.73–0.82	2337
3′ UTR	1.31E-07	1.18	1.10–1.25	1688
AUG (+/– 25 nt)	1.75E-30	2.57	2.19–2.96	203
Brain				
5′ UTR	0.001	1.37	1.13–1.65	123
CDS	1.74E-244	0.11	0.09–0.13	157
3′ UTR	1.12E-208	6.23	5.51–7.21	1028
AUG (+/– 25 nt)	1.98E-19	3.90	3.00–4.99	67

*Significance threshold *p* < 0.05

We then sought to determine if there is a potential location bias within the 5′ UTR, 3′ UTR, and CDS. To this end, meta-gene profiles were generated on normalized rescaled segments of the respective sections. For comparison, the same analyses were performed with Cs sampled randomly from the three segments of the same transcripts (Additional file 2: Figure S7). These analyses revealed a pronounced increase in m5C frequency towards the end of the 5′ UTR and at the very beginning of the CDS in both total poly(A) RNA samples, suggesting enrichment around the translational start codon (Fig. 5b, c, Additional file 2: Figure S7). Indeed, statistical analysis of m5C distribution in the vicinity of the start codon (+/– 25 nt) demonstrated highly significant enrichment of m5C in this region when compared to random C distribution (Table 1). Furthermore, we noted that the distribution of m5C sites in the 3′ UTRs was not uniform in the different transcript categories. Specifically, in transcripts methylated in total poly(A) RNA of both ESCs and brain, we observed increased m5C frequency in the middle of the 3′ UTRs, in transcripts uniquely methylated in the brain, the peak shifted towards the 3′ end, while in transcripts methylated in ESCs only, m5C distribution was flat (Additional file 2: Figure S7).

In summary, we find a previously unknown distinct propensity for m5C to accumulate around the translational start codon in total poly(A) RNA. By contrast, the CDS is depleted of m5C. The 3′ UTRs show a differentiated picture, with clear enrichment for m5C positions found in brain and weak or no enrichment for sites exclusively methylated in ESCs. Thus, cytosine methylation in the 3′ UTR appears to be linked to the cell type as well as to the nature of the transcript.

#### Nuclear poly(A) RNA

Performing the same analyses as described above with the m5Cs detected in the nuclear fraction of poly(A) RNA revealed substantial differences in the m5C distribution pattern in nuclear poly(A) RNA compared to total poly(A) RNA. In both ESCs and brain, the great majority of m5C sites mapped to introns and non-annotated sequences in nuclear RNA. This was particularly pronounced for brain RNA, where 69.9% of all detected m5Cs decorated intronic sequences (ESCs 44.8%). Similar to the poly(A) RNA samples, we found for the mRNA sequences that the relatively largest fraction of m5Cs mapped to the CDS in ESCs and to the 3′ UTR in the brain, respectively (Fig. 5d). Enrichment analysis again revealed significant enrichment of m5Cs in 5′ UTRs, although it was less pronounced than in total poly(A) RNA (Table 1; Fig. 5e, f). In contrast to total RNA, however, m5C sites were weakly enriched in the 3′ UTR of ESCs and strongly enriched in brain mRNAs (Table 1). Also in this case, a location change of the 3′ UTR peak towards the 3′ end was clearly detectable between transcripts methylated in both ESC and brain and those uniquely methylated in the brain. Methylated cytosines were depleted from the CDS as in total poly(A) RNA, except for transcripts uniquely methylated in ESCs, for which a slight enrichment was observed (odds ratio 1.29, *p* = 2.9E-12) (Table 1; Additional file 2: Figure S7). Moreover, the significant enrichment of m5C sites around the translational start codon was also observed in nuclear poly(A) RNA (Table 1), although the peaks were slightly smaller than in total poly(A) RNA (Fig. 5e, f; Additional file 2: Figure S7).

Thus, our analyses reveal distinct m5C localization bias within transcripts of ESCs and the brain. In addition, m5C distribution is different in total poly(A) RNA and nuclear poly(A) RNA, with the latter exhibiting more pronounced accumulation of m5C in the 3′ UTR and less pronounced accumulation in the 5′ UTR. In both nuclear and total poly(A) RNA, the relative distribution of m5C sites within the 3′ UTR correlates with the cell/tissue type as well as with the nature of the transcript.

### Overlap with functionally important motifs

**Fig. 6:**
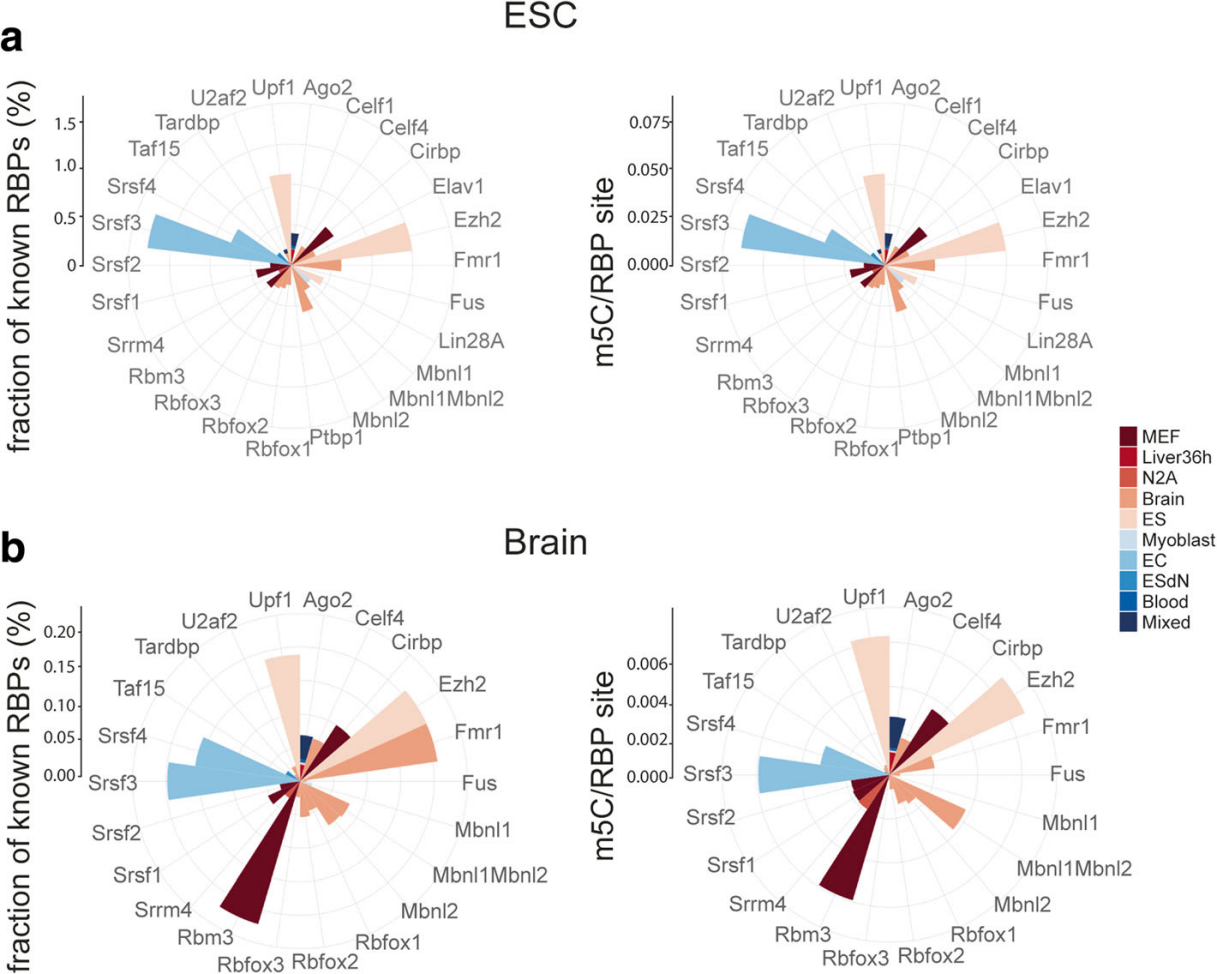
Radar plots show an overlap of m5C sites with binding sites of several RNA binding proteins (*RBPs*) available in the CLIPdb. **a** *Left panel*, fraction of binding sites overlapping with an m5C site for each particular RBP. *Right panel*, number of m5Cs overlapping with binding sites for a particular protein was normalized against the total number of binding sites of the respective RBP. Cell/tissue types in which the RBP binding sites had been detected are color coded and explained in the legend (*MEF* mouse embryonic fibroblasts, *Liver36h* liver partial hepatectomy 36 h, *N2A* Neuro2a, *ES* embryonic stem cells, *EC* embryonal carcinoma, *ESdN* ES-derived neuronal). **b** Same as in **a** for brain total and nuclear poly(A) RNA

We found that brain nuclear and total transcripts in particular show accumulation of m5C sites in the 3′ UTR (Fig. 5). Therefore, and because a previous m5C analysis in human cells found a correlation between Argonaute (Ago) binding sites and m5C position [32], we examined if miRNA binding sites are linked to the m5C mark. To this end, we searched all m5C sites identified in the 3′ UTRs of total poly(A) RNA against the miRNA target sites available at microRNA.org [38]. For comparison, we used an equal number of Cs randomly sampled from the same 3′ UTRs to test for the probability of an overlap between miRNA and m5C sites. Surprisingly, random permutation analysis revealed that m5C sites were depleted rather than enriched at the miRNA target sites (Table 2). We then determined if, perhaps, m5Cs overlap with binding sites of the miRNA binding protein Argonaute 2 (Ago2), and found that although the fraction of Ago2 sites coinciding with m5C was quite low in both ESCs and brain (0.4% and 0.06%, respectively; Fig. 6, Additional file 9), permutation analysis revealed it to be significantly increased compared to random Cs. Nevertheless, in light of the negative correlation between miRNA sites and m5Cs and the very low numbers of overlapping Ago2 binding sites, we conclude that there is no strong link between m5C and miRNA-mediated transcript regulation.

**Table 2:** Overlap of m5Cs with miRNA target sites in the 3′ UTR of ESC and brain RNA

	Total poly(A) RNA		Nuclear poly(A) RNA	
	ESC	Brain	ESC	Brain
m5Cs in 3′ UTR	1774	863	1687	1027
3′ UTRs with m5Cs	700	287	686	282
m5Cs in miRNA targets	310	63	274	84
miRNA targets with m5Cs	241	43	233	62
miRNA targets in 3′ UTRs with m5Cs	13,629	5693	12,050	5535
*p* value (random permutation test)	0.100	10E-4	0.0634	10E-4
Z-score	–1.2454	–5.5334	–1.5002	–5.6833
No. of iterations	10,000	10,000	10,000	10,000

We also analyzed the relationship between other RNA-binding proteins (RBPs) for which data are available in CLIPdb [39] and m5C sites identified in this study. About 29% of m5Cs in ESC and 11% of brain total poly(A) RNA sites overlapped with mapped RBP binding sites. Several RBPs showed statistically significant enrichment of m5C in their binding sites compared to randomly sampled Cs of the same pool of transcripts (Fig. 6, Additional file 9). In particular, the largest relative overlaps were found for UPF1, a protein involved in nonsense-mediated RNA decay, the splicing factors SRSF3 and SRSF4, and the PRC2 subunit EZH2 (Fig. 6, Additional file 9). Collectively, these data suggest that cytosine methylation may be involved in the binding of certain RBPs. Considering the relatively low numbers of RBP sites overlapping with m5C, however, such a potential role may be very specific to a particular transcript rather than a general way to regulate factor binding.

## Discussion

In this study, we present a comparative analysis of cytosine methylation in two mouse cell types/tissues in total and nuclear poly(A) RNAs. We have analyzed undifferentiated pluripotent embryonic stem cells on one hand, and we have examined the brain as a highly differentiated and multi-cell type tissue on the other hand. Using high stringency criteria and independent quality control experiments, we identified m5C sites in several hundred mRNA and in non-coding transcripts, and we show that there are considerable differences in number and distribution of methylated Cs in the different samples. Our data reveal a higher diversity of methylated mRNAs in ESCs compared to brain. The GO analysis showed that transcripts that were methylated exclusively in ESCs or the brain, respectively, were enriched in categories that are characteristic for that particular cell or tissue type. For example, in highly proliferative ESCs that possess very dynamic chromatin, GO terms, such as cell cycle, RNA, and chromatin modification, were enriched among the methylated transcripts, whereas in the brain, methylated transcripts were enriched in categories related to ion transport or synapse function. It is interesting to note that, particularly in ESCs, most of the sites that were methylated specifically in ESCs were not methylated in the brain samples, although the transcripts were expressed. Hence, it is possible that differential methylation of transcripts in different cell types is involved in modulating the properties of a particular transcript with respect to turn-over or translation.

### Cytosine methylation accumulates around the translational start codon

To date, the molecular function of m5C in mRNA is not known; therefore, we can only speculate about the significance of these findings. One clue may derive from the non-random distribution of methylated Cs along the mRNA sequences. For instance, the distinct m5C peak in the vicinity of the translational start codon may suggest that m5C affects the initiation of translation. This might occur by promoting or inhibiting the efficiency of ribosome scanning and start codon detection. Recent in vitro translation experiments with eukaryotic and bacterial translation systems using either templates in which all Cs were replaced by m5C or where m5C was incorporated into a single codon suggest that m5C affects translation in a negative way [40, 41]. Yet, these studies did not address the question of a translation initiation-specific function of m5C. Interestingly, two recent studies reporting the identification of m1A throughout the transcriptome of mammalian and yeast cells showed that m1A is distinctly enriched in the region harboring the translation initiation site [22, 23], and it was found that the m1A modification correlated with higher protein expression [23]. It is therefore possible that m5C and m1A are functionally linked either by acting in concert or by antagonizing each other.

### Distinct 3′ UTR peaks of m5C in different transcript classes

Our data also revealed increased frequency of m5C sites in 3′ UTRs in some transcript classes, which is consistent with previous findings in human HeLa cells [32]. *N*6-methyladenosine also shows enrichment in the 3′ UTR, specifically around the translation stop codon [6, 42]. Comparison with our data, however, revealed that m5C is rather depleted from the m6A peak area at the stop codon (Additional file 2: Figure S8). Instead, we find intriguing differences of the relative locations of the respective m5C peaks in transcripts common to ESCs and brain, ESC-specific ones, and brain-specific ones. These results may suggest different functional roles of cytosine methylation in the different transcript classes. For example, m5C could prevent or promote the binding of miRNAs or of RNA binding proteins (RBPs). Indeed, Squires et al. [32] demonstrated an enrichment of Argonaute I–IV binding sites around 3′ UTR m5Cs in HeLa cells. Our analyses in the mouse also revealed statistically significant enrichment of Ago2 sites around m5Cs; however, the actual fraction of Ago2 binding sites that overlaps with m5C was below 0.5%, and m5C is actually depleted from miRNA target sites. Thus, these data do not clearly point towards a role of m5C in miRNA-mediated regulation. By contrast, we detected slightly higher overlap rates for UPF1, SRSF3 and SRSF4, and the PRC2 subunit EZH2. In an earlier work, using an in vitro assay, we have shown that m5C can interfere with the binding of PRC2 to the A region of the human lncRNA XIST [34]. Thus, it is tempting to speculate that m5C might generally regulate PRC2 binding to its targets. Similarly, m5C could interfere with the binding of other proteins involved in RNA metabolism. Hence, the presence of m5C peaks at different locations in the 3′ UTR may modulate the function of distinct functional mRNA classes in specific ways.

### Increased cytosine methylation frequency in nuclear poly(A) RNA

By comparative analyses of total and nuclear poly(A) RNA fractions, we discovered substantially higher numbers of methylated cytosines in the nuclear fraction with the majority of them mapping to introns and non-annotated regions. This observation raises the possibility that m5C may be involved in the splicing process or may mark transcripts for degradation. Another intriguing possibility is that m5C may decorate regulatory RNAs, such as promoter- or enhancer-derived transcripts [43], which was indeed demonstrated by Aguilo et al. in a recent work [44].

### Bisulfite sequencing as a method to determine global transcriptome methylation

In contrast to the recently developed immunoprecipitation-based techniques, aza-IP [29] and miCLIP [30], which are suitable for identifying the methylation targets of specific RNA methyltransferase (RNMT) enzymes, the BS-seq approach used in this study allows for an unbiased mapping of global cytosine methylation at single nucleotide resolution as well as for determining the extent of methylation of a particular C. However, it is possible that cytosine modifications other than m5C, e.g., 5-hydroxymethylcytosine (hm5C), *N*4-methylcytosine (4mC), 3-methylcytosine, *N*4,2′-*O*-dimethylcytidine (m4mC), or *N*4-acetylcytosine (ac4C), may be resistant to bisulfite-mediated deamination [31]. It was recently shown by mass spectrometry that hm5C is present in poly(A) RNA at a level of ~0.002% of total Cs, while m5C was determined to be in the range of 0.02–0.1% of total Cs [45]. Interestingly, another recent study reported transcriptome-wide mapping of hm5C in *Drosophila melanogaster* using the meRIP method and implied the single fly homolog of the ten-eleven translocation (TET) protein family in its formation [41]. Thus, it is likely that our analysis slightly overestimates the true number of m5C sites, as a few of them might correspond to hm5C or even other cytosine modifications.

There is some discussion in the field as to the actual existence of m5C in mRNA. Available data for targets of the RNA methyltransferases NSUN2 and DNMT2 revealed no or very few modified mRNAs [46, 47]. The fact that there are at least seven other cytosine RNMTs, however, leaves space for the possibility that one of those enzymes or an as yet unidentified enzyme may modify mRNA. One reason for the skepticism about m5C data obtained by bisulfite sequencing lies in the basic reaction mechanism of bisulfite-mediated cytosine modification, which is inhibited by secondary structure [48] and thus may give rise to false-positive callings. To control for this effect, we included spike-in negative controls that correspond to a highly structured region in 16S rRNA, and we have performed three cycles of bisulfite treatment, which serves to progressively destabilize structure. The experiments using RNA oligonucleotides with highly stable secondary structures confirmed that our bisulfite protocol is able to efficiently deal with the structure problem. Importantly, we eliminated all candidate sites from the dataset that were computationally predicted to adopt a base-paired conformation, and we applied high stringency mapping and m5C calling parameters that depend on the analysis of multiple biological replicates. In fact, it is possible that a considerable number of true positives were discarded due to the rigorous filtering. Considering further the positive validation of several target RNAs by meRIP as an alternative method, we think it is reasonable to conclude that we have generated a high-confidence dataset for future studies. Importantly, our study is also supported by several recent studies that clearly demonstrated by mass spectrometry analysis that m5C is present in poly(A) RNA [45, 49, 50].

## Conclusion

In summary, our study presents, to our knowledge, the first comprehensive picture of cytosine methylation in the mouse epitranscriptome and identifies hundreds to thousands of methylation sites in mRNA, yet much fewer in ncRNAs. The data revealed intriguing differences with respect to m5C numbers and position bias between embryonic stem cells and the brain and between total and nuclear poly(A) RNA fractions. One of the next big challenges will be to identify the enzymes that are responsible for targeting specific positions/regions in mRNAs for methylation. Detailed analyses of the candidate RNMTs NSUN2 and DNMT2 have shown that both have a preference for tRNAs and/or more abundant ncRNAs [3, 29–31, 51, 52], and rRNA and tRNAs are also the only identified targets for other studied NSUN proteins to date [53–58]. Therefore, our data provide the foundation for future studies in the mouse, an organism that is highly amenable to experimental manipulation, to address important questions regarding targeting and the functional impact of m5C on the epitranscriptome.

## Methods

### Sample material

Female mouse embryonic stem cells isolated from 129S2/SvPasCrl-derived blastocysts were cultured in ESC-2i/leukemia inhibitory factor (LIF) medium (Dulbecco’s modified Eagle’s medium [DMEM] high glucose with GlutaMAXTM-1 (Gibco), 20% ES cell tested fetal bovine serum (FBS, Gibco), 1 x Non-Essential Amino Acid (NEAA, Gibco), 0.05 mM β-mercaptoethanol, 12.5 mg/L LIF, 3 mM CHIR99021, and 1 mM PD0325901 (both Axon Medchem)). Whole brains were dissected from 7-week-old female 129S2/SvPasCrl mice, rinsed in phosphate-buffered saline (PBS), and snap frozen in liquid nitrogen.

### Preparation of nuclei

1 × 10^7^ mouse ESCs were lysed in hypotonic buffer (10 mM 4-(2-hydroxyethyl)-1-piperazineethanesulfonic acid [HEPES]-KOH, pH 7.9, 10 mM KCl, 1.5 mM MgCl_2_, 0.5 mM dithiothreitol [DTT], 0.5 mM phenylmethanesulfonyl fluoride [PMSF]) and nuclei were collected by centrifugation at 4000 rpm. Adult mouse brain tissue (up to 500 mg) was pulverized in liquid nitrogen using a CryoPrep instrument (Covaris). The frozen powder was resuspended in 2 mL of nuclear extraction buffer (10 mM Tris-HCl, pH 8, 0.32 M sucrose, 5 mM CaCl_2_, 3 mM magnesium acetate, 0.1 mM EDTA, 0.1% Triton X-100 (w/v)) followed by douncing. Nuclei were collected by ultracentrifugation through a sucrose cushion (10 mM Tris-HCl pH 8, 1.8 M sucrose, 3 mM magnesium acetate) at 50,000 rpm for 2.5 h using a SW-55 Ti rotor.

### RNA isolation

RNA was isolated in biological triplicates from pulverized whole brain tissue (up to 250 mg) and 5 × 10^6^ mouse embryonic stem cells as well as from nuclei of both sources using TRIzol (Sigma-Aldrich) following the manufacturer’s recommendations. RNA was treated with 2U of DNase I (New England Biolabs, Ipswich, MA, USA) for 15 min at 37 °C and purified using RNA Clean & Concentrator 25 Kit (Zymo Research, Irvine, CA, USA). Isolated RNA was then subjected to two rounds of poly(A) RNA enrichment using fresh Dynabeads (Ambion) for each round. RNA quality was assessed using an Agilent 2100 Bioanalyzer, and concentration was determined by measuring absorbance at 260 nm and 280 nm in a UV/vis-spectrophotometer.

### Generation of in vitro transcribed spike-in controls

The regions spanning nt 914–1465 of *E. coli* 16S rRNA and the entire pET15b vector sequence (New England Biolabs) were used as templates for in vitro transcription. First, the region of interest of *E. coli* 16S rRNA was amplified by PCR with a forward primer harboring a T7 promoter sequence (Additional file 10). The pET15b vector was linearized by *Bgl*II and gel purified. We used 1 μg of PCR product or 500 ng of pET15b vector for in vitro transcription with a MEGAScript Kit (Ambion) according to the manufacturer’s protocol. In vitro transcribed RNA was treated with 1 μL TURBO DNase I (2 U/μL) for 15 min at 37 °C to remove residual DNA template and subsequently precipitated by adding 1 volume of 7.5 M LiCl. The pellet was dissolved in RNase-free a.d. provided in the kit and denatured at 70 °C for 30 min in an Eppendorf incubator. Subsequently, the RNA was left to refold during slow cooling to room temperature in the switched-off incubator. The refolded in vitro transcribed controls were added to the RNA samples before bisulfite treatment at a mass ratio of 1:20,000.

### Bisulfite treatment

Bisulfite treatment was performed as described previously [34] using the EZ RNA methylation Kit (Zymo Research). Briefly, 1–2 μg poly(A) RNA was converted using three cycles of 10-min denaturation at 70 °C followed by 45 min at 64 °C. RNA separation from bisulfite solution, desulfonation, and purification were performed using the kit. RNA quantity was determined by absorbance measurement at 260 nm using a Nanodrop UV/Vis spectrophotometer (PeqLab). The efficiency of the bisulfite treatment was tested by PCR-mediated bisulfite analysis [34] of the spiked-in non-methylated control sequences.

### Library preparation and sequencing

Sequencing libraries were prepared using the ScriptSeq V2 RNA-Seq Library Preparation Kit (Epicentre), purified with AMPure XP beads (Beckman Coulter) and quantified using the KAPA Library Quantification Kit for Illumina platforms (KAPA Biosystems). RNA fragmenting was omitted, as bisulfite treatment results in fragmentation to 100–250 nt. Libraries were multiplexed at 11 pM and sequenced on an Illumina HiSeq 1500 platform using 100 bp single-end reads in the case of ESC, and paired-end reads in the case of mouse brain. Sequencing runs generated >70 million reads per sample.

### Mapping of sequencing reads and m5C calling

Raw sequencing data were extensively filtered to remove low-quality reads and adapter contaminations. Clean reads were mapped to the mouse genome GRCm38/mm10 using the splice-aware RNA-BSseq alignment tool meRanGs available with meRanTK version 1.0 [35]. Unambiguously aligned reads were then used to call candidate m5Cs using meRanCall from meRanTK version 1.0 (FDR <0.01). Based on the m-bias plots (Additional file 2: Figure S9) obtained from meRanGs, 10 bases on the 5′ end of forward reads and 7 bases on the 5′ end of reverse reads were excluded from methylation calling. Furthermore, only bases with a base-call quality score of *Q* > =35 for single-end reads and *Q* > =30 for paired-end reads were considered for methylation calling. Candidate cytosine positions were covered by at least 10 reads and had a conversion rate less than 0.8. An m5C candidate had to be present in all three replicates of a given sample. Subsequently, the full-length transcripts containing an m5C candidate were extracted from the RefSeq database (GRCm38.p3) and subjected to secondary structure analysis using RNAfold of the ViennaRNA package (version 2.2.8) [59]. We calculated the maximum expected accuracy (MEA) structure at 70 °C (the temperature of the bisulfite conversion reaction) using a gamma of 0.1. The maximum allowed distance between two bases in a pair was set to 150 nt to keep a reasonable computation time. For introns and non-annotated sequences, 300 nt around the m5C candidate position were subjected to folding analysis. Only candidate m5C sites that were predicted not to be base-paired in the resulting structure were retained. The final lists of candidate m5Cs (Additional files 4, 5, 7, and 8) as well as the lists of called m5Cs in the individual replicates prior to additional filtering were uploaded to the GEO database [GEO:GSE83432]. The latter list also shows the identity of the base on the reference genome for each m5C candidate to allow for identification of potential SNPs. The number of detected SNPs was ≤5 in the compiled replicates of total poly(A) RNA and ≤17 for nuclear poly(A) RNA. Thus, SNPs do not pose a problem for m5C calling. To compile lists of unique and common m5Cs, meRanCompare (1.0) was used [35]. An m5C was considered *unique* to a sample type if it was found in three replicates in one sample type (e.g., ESC total RNA) but not in any one of the replicates of the other sample type (e.g., brain total RNA). The m5C candidates considered *common* to two sample types were present in three replicates of one sample and in at least one replicate of the other sample. To analyze a potential link between differential methylation and expression in the *unique* groups, we determined for all m5Cs classified as unique in one sample type the expression of the corresponding gene, the sequencing read coverage of the respective m5C, and also its non-conversion rate in all three replicates of the other sample type. To this end, we used the methylation calling procedure described above. Genes were classified as expressed if the corresponding transcript sequencing coverage exceeded a mean normalized count of 10. If the *unique* C of one sample type was covered by <10 reads (mean normalized count) in the other sample, it was designated as “low position coverage.” If the *unique* C of one sample type was covered by >10 reads in the other sample but the mean non-conversion rate was <0.2, it was designated as “low methylation rate.” Positions in which the mean values for coverage and non-conversion were skewed towards methylation by an individual replicate were classified as “biased mean.”

### Analysis of m5C position bias

To assess a possible positional bias of m5Cs, mRNAs from the RefSeq database (GRCm38.p2) were divided into three segments (5′ UTR, CDS, 3′ UTR). Each segment was normalized according to its average length. The total normalized transcript was binned into regions of 1% of the total length, and the percentage of m5Cs contained in each bin was calculated and plotted along a normalized meta-gene. To test for enrichment or depletion in each of the segments, a two-sided Fisher’s exact test was used in which all possible cytosine positions in each of the three segments (5′ UTR, CDS, 3′ UTR) of the transcripts with an observed m5C were considered. In addition to these three main segments, the +/– 25 nt region around the AUG (translation start site) was tested in the same way. In parallel, a set of random Cs (corresponding to the number of observed m5Cs) was picked from the same transcripts and analyzed identically to produce and visualize random meta-gene profiles.

### Comparison with miRNA target sites

Conserved miRNA target sites from the mouse with mirSVR score < –0.1 were downloaded from microRNA.org [38], transferred to mm10, and unique miRNA target sites were overlapped with m5Cs found in 3′ UTRs. To test the statistical significance of the number of observed overlaps, a permutation test was used, in which a number of Cs equal to the number of m5Cs observed in miRNA target sites was randomly sampled from all possible Cs in 3′ UTRs that contained an observed m5C. The test was run for 10,000 iterations, and the resulting *p* values and Z-scores were reported.

### Comparison with CLIPdb data

Binding sites of murine RNA binding proteins reported by PiRaNhA [60] were downloaded from CLIPdb [39]. All available binding sites from all factors and cell types were combined into one BED file, and sites closer to each other than 15 nt were merged. From these, only sites that were unique to a given RNA binding protein and that were shorter than 200 nt were considered for further analysis. The fraction of total known RNA binding protein (RBP) sites overlapping with an m5C site and the number of m5C sites per RBP site was calculated for each RBP and cell type in which the RBP was reported and plotted as bar plots in polar coordinates (radar plots). To test significance of the observed overlaps between m5Cs and binding sites of individual RBPs, a permutation test was used in which a number of Cs equal to the number of m5Cs overlapping with the RBP sites was randomly sampled from all possible Cs in transcripts that had a mean normalized read count greater than 10. The test was run for 10,000 iterations, and the resulting *p* values and Z-scores were reported.

### Estimation of gene expression

Gene expression was estimated by using the BAM files containing meRanGs-mapped reads. Read counts over exons were obtained by HTSeq [61], and gene expression was determined by DESeq2 [62].

### GO analysis

Gene Ontology (GO) analysis was performed by entering gene IDs of transcripts found to be methylated in the total poly(A) RNA samples into the Gene Functional Annotation Tool at the DAVID website (http://david.abcc.ncifcrf.gov/; version 6.8 Beta). Input was defined as gene list, and official gene symbols were used as identifiers. As a background for the analyses of transcripts methylated uniquely in ESCs or brain, we used the lists of expressed genes (mean normalized read count >10) in ESCs or brain, respectively. For transcripts methylated in both samples, all genes expressed in both samples (>10 reads) were used. Predefined parameters were used for the enrichment analysis for biological process, molecular function, and cellular component. Resulting GO terms and the corresponding *p* values were then processed by using REVIGO [63], a tool that summarizes long lists of GO terms by removing redundant ones. The allowed similarity was set to 0.5 in the REVIGO settings. The ten most significant categories were shown.

### Comparison of m5C and m6A distribution around translational start and stop codons

All available mouse m6A peaks obtained with MACS2 (https://pypi.python.org/pypi/MACS2) [64] were downloaded from MeT-DB [65] and combined into a single BED file with unique peaks. The resulting BED file was transferred from mm9 to mm10, and peak sequences were extracted from the mm10 genome. These sequences were then scanned with Find Individual Motif Occurrences (FIMO) available with the MEME software suite [66] using a position weight matrix representing the canonical m6A motif (HGGACNN) [67]. All unique m6A motif site locations were then compared with m5C sites unique to ESC or brain and common to ESC and brain (all for total and nuclear poly(A) RNA). m5C and m6A site locations within +/– 500 nt of the AUG and STOP codons were plotted in 25-nt bins as percentages of all modified m5Cs or m6A sites.

### Immuno-northern blot

For immuno-northern blotting, in vitro transcripts corresponding to nt 914–1465 of *E. coli* 16S rRNA were generated and purified as described above using the MEGAScript Kit (Ambion) according to the manufacturer’s protocol with the following modification: in vitro transcription reactions were supplemented with 5-methylcytidine-5’-triphosphate (Trilink Biotechnologies, San Diego, CA, USA) to obtain a ratio of 0%, 50%, and 100% to cytidine-5’-triphosphate. After purification as described above, 1 μg of the transcripts was denatured by incubation at 65 °C for 10 min, electrophoresed on a denaturating 1.2% agarose gel, and blotted onto a Hybond-N nylon membrane (GE Healthcare). Blotted RNA was cross-linked in a Stratalinker 2400 UV Crosslinker at 1200 μJ UV with auto-cross-linking setting. The membrane was washed three times for 10 min in 0.1X SSC (1X SSC: 150 mM NaCl, 15 mM sodium citrate, pH 7) and blocked for 1 h in 1xBlocking Buffer (10X Blocking Buffer: 10% (w/v) Blocking Reagent (Roche) in Buffer P1; Buffer P1: 100 mM maleic acid, 150 mM NaCl, pH 7) at room temperature. Incubation with anti-m5C antibody (Diagenode, MAb-081-100) was performed for 3 h at room temperature with a 1:500 dilution of the antibody in Blocking Buffer. Subsequently, blots were washed three times for 10 min in 0.1X SSC and incubated with secondary antibody (1:10,000, anti-mouse light-chain specific secondary antibody, Jackson ImmunoResearch) in Blocking Buffer for 1 h at room temperature. After three washes in 0.1X SSC, membranes were washed twice in Tris-buffered saline with Tween (TBST), chemiluminescence was developed using ECL Prime Western Blotting Detection Reagent (GE Healthcare), and signals were detected in a Fusion SL 3500 WL (Vilber).

### Methylation-RNA immunoprecipitation (meRIP)

Isolated RNA was randomly fragmented by incubation at 75 °C for 3 min using 1X fragmentation buffer (10 mM Tris-HCl pH 7, 10 mM ZnCl_2_). Fragmentation was stopped by adding 1X Stop Solution (0.05 M EDTA). We incubated 2 μL of anti-m5C antibody (2 mg/mL; Diagenode, MAb-081-100) with 30 μL of protein G sepharose (GE Healthcare) in 300 μL IP buffer (10 mM Tris-HCl pH 7.5, 150 mM NaCl, 0.05% Triton-X (v/v)) with 2 μg of random 25 nt oligonucleotides to reduce unspecific binding for 2 h at 4 °C on a rotating wheel. The same procedure was performed for a control reaction using mouse IgGs (Santa Cruz Biotechnology). Bead-antibody complexes were washed three times with IP buffer and finally brought to 250 μL with IP buffer and supplemented with 200 ng control RNA (in vitro transcribed *E. coli* 16S rRNA nt 914–1125). A 10-μg sample of RNA was added to the bead-antibody complexes and incubated with 1 μL RNasin overnight at 4 °C on a rotating wheel. After several washes with IP buffer, RNA was incubated in 300 μL elution buffer (5 mM Tris-HCl pH 7.5, 1 mM EDTA, 0.05% SDS, and 80 μg Proteinase K) for 1 h at 50 °C. Beads were removed by centrifugation in a microcentrifuge, and the supernatant was mixed with 800 μL TRIzol (Sigma-Aldrich) for RNA isolation as described above. We used 1 μL glycogen (20 μg/μL) as a carrier in the final precipitation step. The RNA pellet was dissolved in 10 μL a.d. and subjected to reverse transcription. Enrichment of candidate RNAs was measured by quantitative real-time PCR of immunoprecipitated RNA by comparing the anti-m5C antibody sample with the IgG control. *E. coli* in vitro transcripts served as an internal unspecific binding control and were used to normalize binding of the RNA of interest to IgG control and the test antibody sample. Data were expressed as relative enrichment over IgG control, and statistical significance was determined by unpaired *t* test of three independent experiments with *p* < 0.05 using GraphPad Prism 7.0. Sequences of primers used for qPCR are shown in Additional file 10.

### RNA synthesis and mass spectrometry analysis

RNA oligonucleotides were synthesized by the solid-phase method as described previously [68]. Purified oligos were denatured at 95 °C for 30 s, refolded in the presence of 100 mM KCl by slowly cooling down to room temperature, and treated with bisulfite as described above. Treated and untreated RNA oligos were analyzed by liquid chromatography-mass spectrometry (LC-MS) as in [68].

## Additional files

Additional file 1: Table S1. General sequencing and m5C calling results. (XLSX 34 kb)
Additional file 2: Figure S1. Distribution of sequencing reads mapping with 0, 1, 2, 3, 4, or 5 mismatches to the reference genome. **Figure S2.** Distribution of cytosine conversion along *E. coli* 16S rRNA negative control. **Figure S3.** Distribution of cytosine conversion along pET15b negative control. **Figure S4.** Mass spectrometry analysis of three unmethylated RNA oligos before and after bisulfite treatment. **Figure S5.** Immuno-northern blot detection of m5C. **Figure S6.** Analysis of uniquely methylated cytosines in nuclear poly(A) RNA of ESCs and brain. **Figure S7.** Meta-gene profiles of m5C distribution in total and nuclear poly(A) RNA from ESCs and brain. **Figure S8.** Distribution of m5C and m6A around translational start and stop codons. **Figure S9.** Cytosine 5 methylation bias (m-bias) plots. (PDF 4485 kb)
Additional file 3: Table S2. C → T conversion rates for spike-in controls. (XLSX 36 kb)
Additional file 4: Table S3. Methylated cytosines in total poly(A) ESC RNA. (XLSX 761 kb)
Additional file 5: Table S4. Methylated cytosines in total poly(A) brain RNA. (XLSX 222 kb)
Additional file 6: Table S5. Total normalized (DEseq2) sequencing counts of total poly(A) RNA from ESC and brain. (XLSX 2456 kb)
Additional file 7: Table S6. Methylated cytosines in ESC nuclear poly(A) RNA. (XLSX 1227 kb)
Additional file 8: Table S7. Methylated cytosines in brain nuclear poly(A) RNA. (XLSX 797 kb)
Additional file 9: Table S8. Statistical analysis of m5C overlap with RNA binding protein (*RBP*) sequences in total poly(A) RNA of ESCs and brain. (XLSX 41 kb)
Additional file 10: Table S9. Primer sequences. (XLSX 25 kb)

## References

[1] Machnicka MA, Olchowik A, Grosjean H, Bujnicki JM (2014). Distribution and frequencies of post-transcriptional modifications in tRNAs. RNA Biol..

[2] Motorin Y, Helm M (2011). RNA nucleotide methylation. Wiley Interdiscip Rev RNA..

[3] Sibbritt T, Patel HR, Preiss T (2013). Mapping and significance of the mRNA methylome. Wiley Interdiscip Rev RNA..

[4] Meyer KD, Jaffrey SR (2014). The dynamic epitranscriptome: N6-methyladenosine and gene expression control. Nat Rev Mol Cell Biol..

[5] He C (2010). Grand challenge commentary: RNA epigenetics?. Nat Chem Biol..

[6] Meyer KD, Saletore Y, Zumbo P, Elemento O, Mason CE, Jaffrey SR (2012). Comprehensive analysis of mRNA methylation reveals enrichment in 3′ UTRs and near stop codons. Cell..

[7] Saletore Y, Meyer K, Korlach J, Vilfan ID, Jaffrey S, Mason CE (2012). The birth of the Epitranscriptome: deciphering the function of RNA modifications. Genome Biol..

[8] Jia G, Fu Y, He C (2013). Reversible RNA adenosine methylation in biological regulation. Trends Genet..

[9] Schwartz S (2016). Cracking the epitranscriptome. RNA..

[10] Li S, Mason CE (2014). The pivotal regulatory landscape of RNA modifications. Annu Rev Genomics Hum Genet..

[11] Liu N, Pan T (2015). RNA epigenetics. Transl Res..

[12] Fu Y, Dominissini D, Rechavi G, He C (2014). Gene expression regulation mediated through reversible m^6^A RNA methylation. Nat Rev Genet..

[13] Wang X, Lu Z, Gomez A, Hon GC, Yue Y, Han D (2014). N6-Methyladenosine-dependent regulation of messenger RNA stability. Nature..

[14] Schwartz S, Mumbach MR, Jovanovic M, Wang T, Maciag K, Bushkin GG (2014). Perturbation of m6A writers reveals two distinct classes of mRNA methylation at internal and 5′ sites. Cell Rep..

[15] Geula S, Moshitch-Moshkovitz S, Dominissini D, Mansour AA, Kol N, Salmon-Divon M (2015). Stem cells. m6A mRNA methylation facilitates resolution of naïve pluripotency toward differentiation. Science.

[16] Wang X, Zhao BS, Roundtree IA, Lu Z, Han D, Ma H (2015). N(6)-Methyladenosine modulates messenger RNA translation efficiency. Cell..

[17] Liu N, Dai Q, Zheng G, He C, Parisien M, Pan T (2015). N(6)-Methyladenosine-dependent RNA structural switches regulate RNA-protein interactions. Nature..

[18] Zhao X, Yang Y, Sun B-F, Shi Y, Yang X, Xiao W (2014). FTO-dependent demethylation of N6-methyladenosine regulates mRNA splicing and is required for adipogenesis. Cell Res..

[19] Batista PJ, Molinie B, Wang J, Qu K, Zhang J, Li L (2014). m(6)A RNA modification controls cell fate transition in mammalian embryonic stem cells. Cell Stem Cell.

[20] Carlile TM, Rojas-Duran MF, Zinshteyn B, Shin H, Bartoli KM, Gilbert WV (2014). Pseudouridine profiling reveals regulated mRNA pseudouridylation in yeast and human cells. Nature..

[21] Schwartz S, Bernstein DA, Mumbach MR, Jovanovic M, Herbst RH, León-Ricardo BX (2014). Transcriptome-wide mapping reveals widespread dynamic-regulated pseudouridylation of ncRNA and mRNA. Cell..

[22] Li X, Xiong X, Wang K, Wang L, Shu X, Ma S (2016). Transcriptome-wide mapping reveals reversible and dynamic N(1)-methyladenosine methylome. Nat Chem Biol..

[23] Dominissini D, Nachtergaele S, Moshitch-Moshkovitz S, Peer E, Kol N, Ben-Haim MS (2016). The dynamic N(1)-methyladenosine methylome in eukaryotic messenger RNA. Nature..

[24] Salditt-Georgieff M, Jelinek W, Darnell JE, Furuichi Y, Morgan M, Shatkin A (1976). Methyl labeling of HeLa cell hnRNA: a comparison with mRNA. Cell..

[25] Dubin DT, Taylor RH (1975). The methylation state of poly A-containing messenger RNA from cultured hamster cells. Nucleic Acids Res..

[26] Desrosiers R, Friderici K, Rottman F (1974). Identification of methylated nucleosides in messenger RNA from Novikoff hepatoma cells. Proc Natl Acad Sci U S A..

[27] Perry RP, Kelley DE, Friderici K, Rottman F (1975). The methylated constituents of L cell messenger RNA: evidence for an unusual cluster at the 5′ terminus. Cell..

[28] Motorin Y, Lyko F, Helm M (2010). 5-methylcytosine in RNA: detection, enzymatic formation and biological functions. Nucleic Acids Res..

[29] Khoddami V, Cairns BR (2013). Identification of direct targets and modified bases of RNA cytosine methyltransferases. Nat Biotechnol..

[30] Hussain S, Sajini AA, Blanco S, Dietmann S, Lombard P, Sugimoto Y (2013). NSun2-mediated cytosine-5 methylation of vault noncoding RNA determines its processing into regulatory small RNAs. Cell Rep..

[31] Schaefer M, Pollex T, Hanna K, Lyko F (2009). RNA cytosine methylation analysis by bisulfite sequencing. Nucleic Acids Res..

[32] Squires JE, Patel HR, Nousch M, Sibbritt T, Humphreys DT, Parker BJ (2012). Widespread occurrence of 5-methylcytosine in human coding and non-coding RNA. Nucleic Acids Res..

[33] Edelheit S, Schwartz S, Mumbach MR, Wurtzel O, Sorek R. Transcriptome-wide mapping of 5-methylcytidine RNA modifications in bacteria, archaea, and yeast reveals m5C within archaeal mRNAs. de Crécy-Lagard V, editor. PLoS Genet. 2013;9:e1003602. 10.1371/journal.pgen.1003602 PMC3694839 23825970

[34] Amort T, Soulière MF, Wille A, Jia X-Y, Fiegl H, Wörle H (2013). Long non-coding RNAs as targets for cytosine methylation. RNA Biol..

[35] Rieder D, Amort T, Kugler E, Lusser A, Trajanoski Z (2016). meRanTK: methylated RNA analysis ToolKit. Bioinformatics.

[36] Vasilyev N, Polonskaia A, Darnell JC, Darnell RB, Patel DJ, Serganov A (2015). Crystal structure reveals specific recognition of a G-quadruplex RNA by a β-turn in the RGG motif of FMRP. Proc Natl Acad Sci U S A..

[37] Duszczyk MM, Wutz A, Rybin V, Sattler M (2011). The Xist RNA A-repeat comprises a novel AUCG tetraloop fold and a platform for multimerization. RNA..

[38] Betel D, Wilson M, Gabow A, Marks DS, Sander C (2008). The microRNA.org resource: targets and expression. Nucleic Acids Res.

[39] Yang Y-CT, Di C, Hu B, Zhou M, Liu Y, Song N (2015). CLIPdb: a CLIP-seq database for protein-RNA interactions. BMC Genomics..

[40] Hoernes TP, Clementi N, Faserl K, Glasner H, Breuker K, Lindner H (2016). Nucleotide modifications within bacterial messenger RNAs regulate their translation and are able to rewire the genetic code. Nucleic Acids Res..

[41] Delatte B, Wang F, Ngoc LV, Collignon E, Bonvin E, Deplus R (2016). RNA biochemistry. Transcriptome-wide distribution and function of RNA hydroxymethylcytosine. Science.

[42] Dominissini D, Moshitch-Moshkovitz S, Schwartz S, Salmon-Divon M, Ungar L, Osenberg S (2012). Topology of the human and mouse m6A RNA methylomes revealed by m6A-seq. Nature..

[43] Fort A, Yamada D, Hashimoto K, Koseki H, Carninci P (2015). Nuclear transcriptome profiling of induced pluripotent stem cells and embryonic stem cells identify non-coding loci resistant to reprogramming. Cell Cycle..

[44] Aguilo F, Li S, Balasubramaniyan N, Sancho A, Benko S, Zhang F (2016). Deposition of 5-methylcytosine on enhancer RNAs enables the coactivator function of PGC-1α. Cell Rep..

[45] Huber SM, van Delft P, Mendil L, Bachman M, Smollett K, Werner F (2015). Formation and abundance of 5-hydroxymethylcytosine in RNA. ChemBioChem..

[46] Hussain S, Aleksic J, Blanco S, Dietmann S, Frye M (2013). Characterizing 5-methylcytosine in the mammalian epitranscriptome. Genome Biol..

[47] Khoddami V, Cairns BR (2014). Transcriptome-wide target profiling of RNA cytosine methyltransferases using the mechanism-based enrichment procedure Aza-IP. Nat Protoc..

[48] Shapiro R, Braverman B, Louis JB, Servis RE (1973). Nucleic acid reactivity and conformation. II. Reaction of cytosine and uracil with sodium bisulfite. J Biol Chem.

[49] Fu L, Guerrero CR, Zhong N, Amato NJ, Liu Y, Liu S (2014). Tet-mediated formation of 5-hydroxymethylcytosine in RNA. J Am Chem Soc..

[50] Huang W, Lan M-D, Qi C-B, Zheng S-J, Wei S-Z, Yuan B-F (2016). Chemical science. R Soc Chem..

[51] Tuorto F, Liebers R, Musch T, Schaefer M, Hofmann S, Kellner S (2012). RNA cytosine methylation by Dnmt2 and NSun2 promotes tRNA stability and protein synthesis. Nat Struct Mol Biol..

[52] Blanco S, Frye M (2014). Role of RNA methyltransferases in tissue renewal and pathology. Curr Opin Cell Biol..

[53] Schosserer M, Minois N, Angerer TB, Amring M, Dellago H, Harreither E (2015). Methylation of ribosomal RNA by NSUN5 is a conserved mechanism modulating organismal lifespan. Nat Commun..

[54] Haag S, Sloan KE, Ranjan N, Warda AS, Kretschmer J, Blessing C (2016). NSUN3 and ABH1 modify the wobble position of mt‐tRNA Met to expand codon recognition in mitochondrial translation. EMBO J..

[55] Haag S, Warda AS, Kretschmer J, Günnigmann MA, Höbartner C, Bohnsack MT (2015). NSUN6 is a human RNA methyltransferase that catalyzes formation of m5C72 in specific tRNAs. RNA..

[56] Metodiev MD, Spåhr H, Loguercio Polosa P, Meharg C, Becker C, Altmueller J (2014). NSUN4 is a dual function mitochondrial protein required for both methylation of 12S rRNA and coordination of mitoribosomal assembly. PLoS Genet..

[57] Nakano S, Suzuki T, Kawarada L, Iwata H, Asano K, Suzuki T (2016). NSUN3 methylase initiates 5-formylcytidine biogenesis in human mitochondrial tRNA(Met). Nat Chem Biol..

[58] Van Haute L, Dietmann S, Kremer L, Hussain S, Pearce SF, Powell CA (2016). Deficient methylation and formylation of mt-tRNA(Met) wobble cytosine in a patient carrying mutations in NSUN3. Nat Commun..

[59] Lorenz R, Bernhart SH, Höner Zu Siederdissen C, Tafer H, Flamm C, Stadler PF (2011). ViennaRNA Package 2.0.. Algorithms Mol Biol..

[60] Murakami Y, Spriggs RV, Nakamura H, Jones S (2010). PiRaNhA: a server for the computational prediction of RNA-binding residues in protein sequences. Nucleic Acids Res..

[61] Anders S, Pyl PT, Huber W (2015). HTSeq—a Python framework to work with high-throughput sequencing data. Bioinformatics..

[62] Love MI, Huber W, Anders S (2014). Moderated estimation of fold change and dispersion for RNA-seq data with DESeq2. Genome Biol..

[63] Supek F, Bošnjak M, Škunca N, Šmuc T. REVIGO summarizes and visualizes long lists of gene ontology terms. PLoS ONE. 2011;e21800. 10.1371/journal.pone.0021800 PMC3138752 21789182

[64] Zhang Y, Liu T, Meyer CA, Eeckhoute J, Johnson DS, Bernstein BE (2008). Model-based analysis of ChIP-Seq (MACS). Genome Biol..

[65] Liu H, Flores MA, Meng J, Zhang L, Zhao X, Rao MK (2015). MeT-DB: a database of transcriptome methylation in mammalian cells. Nucleic Acids Res..

[66] Grant CE, Bailey TL, Noble WS (2011). FIMO: scanning for occurrences of a given motif. Bioinformatics..

[67] Chen K, Lu Z, Wang X, Fu Y, Luo G-Z, Liu N (2015). High-resolution N(6) -methyladenosine (m(6) A) map using photo-crosslinking-assisted m(6) a sequencing. Angew Chem Int Ed Engl.

[68] Riml C, Micura R (2016). Synthesis of 5-hydroxymethylcytidine- and 5-hydroxymethyl-uridine-modified RNA. Synthesis (Stuttg).

