# Supplementary material for: Distinct 5-methylcytosine profiles in poly(A) RNA from mouse embryonic stem cells and brain
Source: Genome Biol. 2017 Jan 5;18:1. doi: 10.1186/s13059-016-1139-1 (PMC5225599; doi:10.1186/s13059-016-1139-1)
Supplement: Additional file 2: Figure S1. — Distribution of sequencing reads mapping with 0, 1, 2, 3, 4, or 5 mismatches to the reference genome. Figure S2. Distribution of cytosine conversion along E. coli 16S rRNA negative control. Figure S3. Distribution of cytosine conversion along pET15b negative control. Figure S4. Mass spectrometry analysis of three unmethylated RNA oligos before and after bisulfite treatment. Figure S5. Immuno-northern blot detection of m5C. Figure S6. Analysis of uniquely methylated cytosines in nuclear poly(A) RNA of ESCs and brain. Figure S7. Meta-gene profiles of m5C distribution in total and nuclear poly(A) RNA from ESCs and brain. Figure S8. Distribution of m5C and m6A around translational start and stop codons. Figure S9. Cytosine 5 methylation bias (m-bias) plots. (PDF 4485 kb) [file 13059_2016_1139_MOESM2_ESM.pdf]

Distinct 5-methylcytosine profiles in poly(A)RNA from mouse embryonic stem cells and the brain

Thomas Amort[1§,] Dietmar Rieder[2§], Alexandra Wille[1], Daria Khokhlova-Cubberley[3], Christian Riml[4], Lukas Trixl[1], Xi-Yu Jia[3], Ronald Micura[4], Alexandra Lusser[1*]

Additional file 2: Supplementary Figures

Supplementary Figures

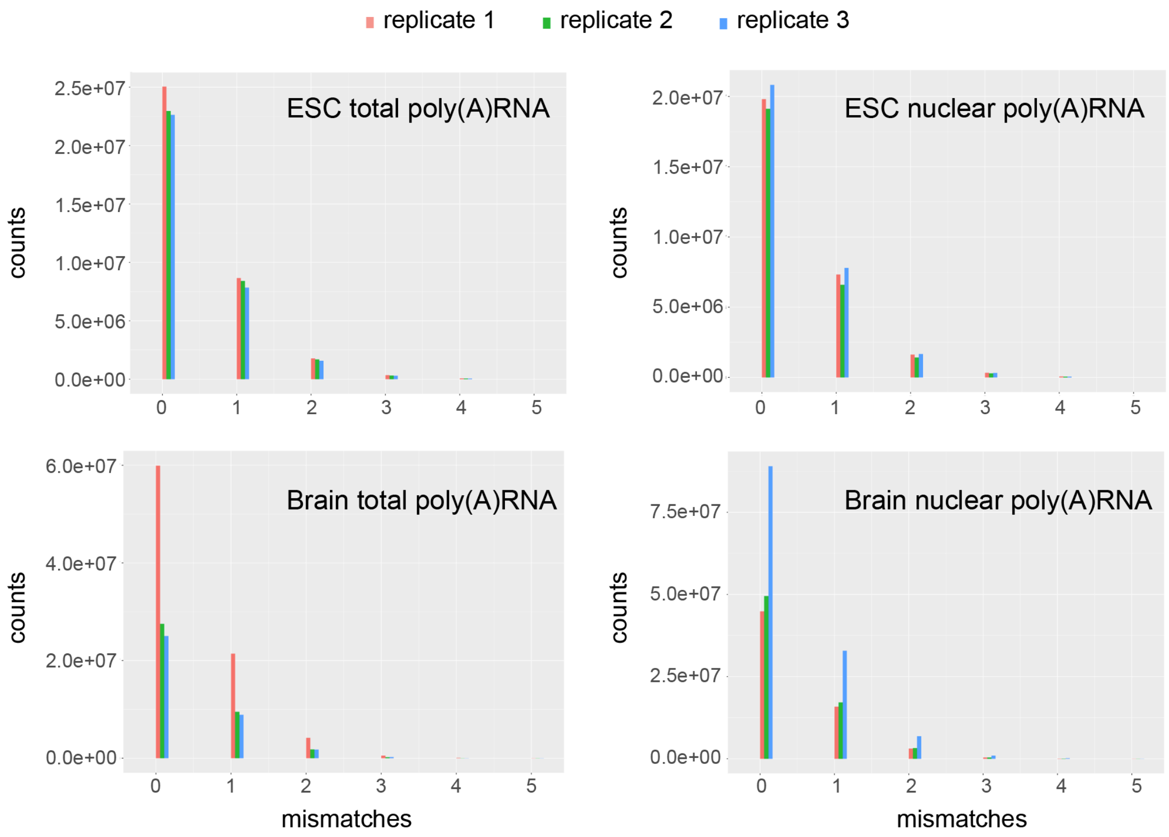

Figure S1. Distribution of sequencing reads aligned with 0, 1, 2, 3, 4 or 5 mismatches to the reference genome. Y-axis represents the number of reads aligned with the number of mismatches indicated on the X-axis.

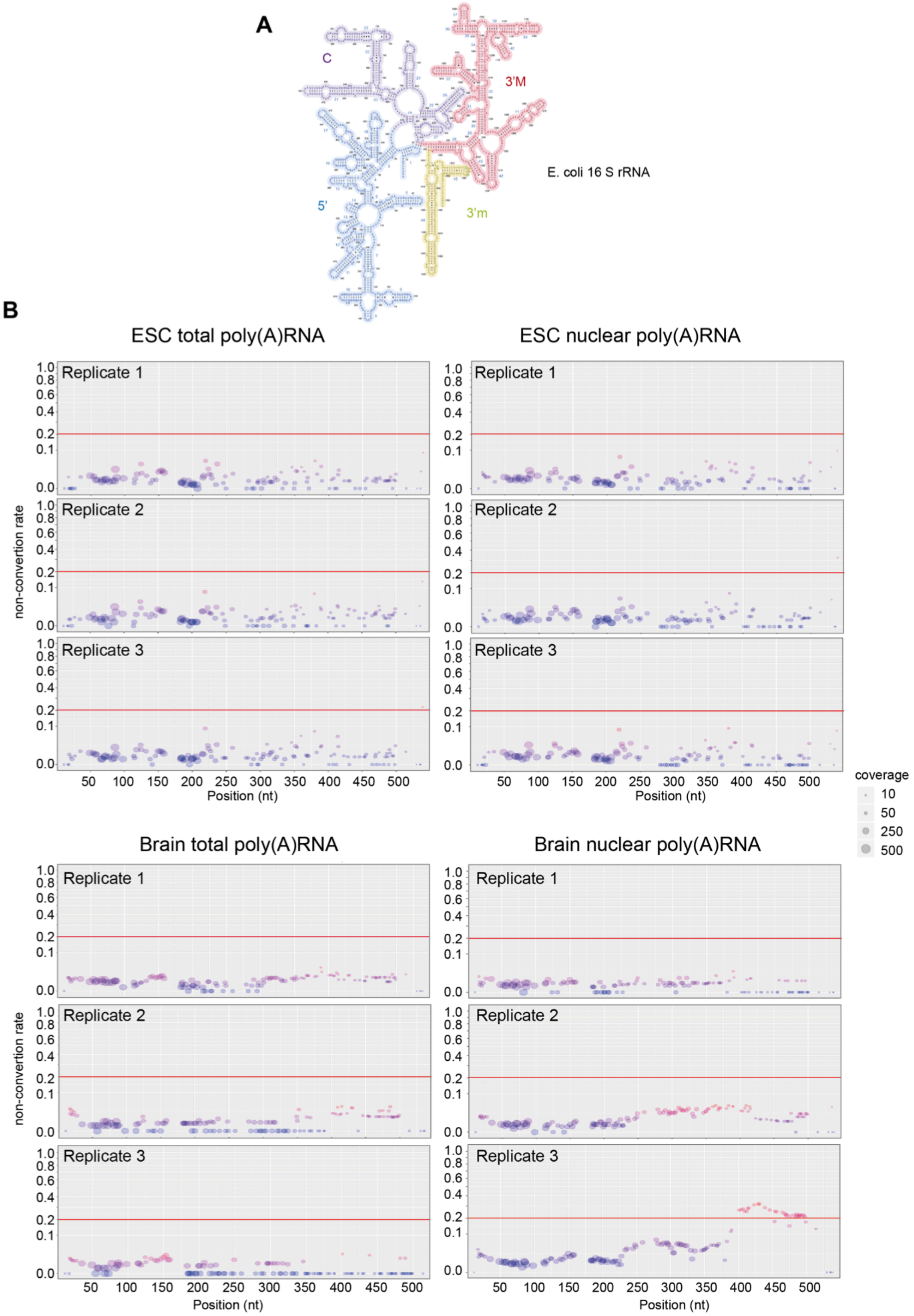

Figure S2. Distribution of cytosine conversion along *E. coli* 16S rRNA negative control. a Secondary structure depiction of *E. coli* 16S rRNA. The spike-in control sequence covers all of region 3'M and half of region 3'm. **b** Plots of non-conversion rates of each cytosine along the 16S rRNA control sequence. The red line marks the non-conversion threshold of 0.2. None of the cytosines shows

non-conversion above 0.2, except for a few in replicate 3 of brain nuclear poly(A)RNA. Importantly, these cytosines were eliminated by filtering for presence in all three replicates. Extend of sequencing coverage is symbolized by circle size and explained in the legend.

A

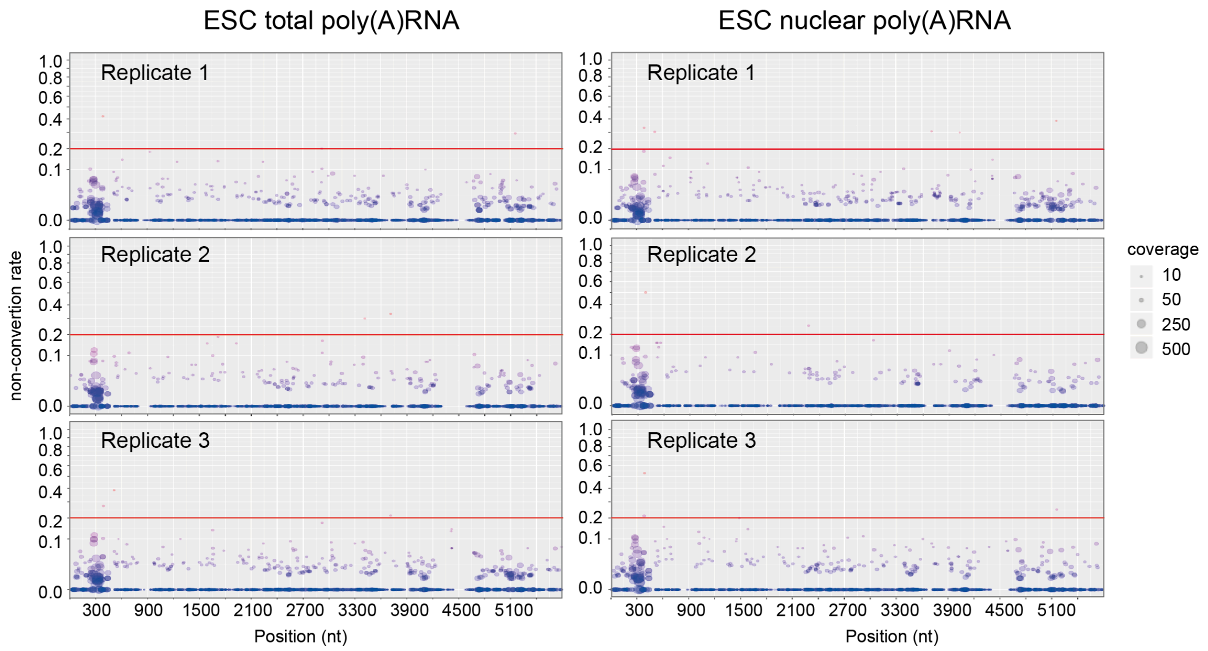

B

refPos	norm(avg/cov)	norm (avg/C_count)	avg/methRate	sem/methRate	comb/p-value mRate	rep
**total poly(A)RNA**						
3713*	16	3	0.249	0.034	7.89E-10	3
**nuclear poly(A)RNA**						
387*	20	9	0.454	0.05	3.84E-07	3

Figure S3. Distribution of cytosine conversion along pET15b negative control. a Plot of non-conversion rates for each cytosine along the entire control sequence. The red line marks the non-conversion threshold of 0.2. Extend of sequencing coverage is symbolized by circle size and explained in the legend. **b** One cytosine each for total and nuclear poly(A)RNA was detected as m5C candidate upon basic data filtering. Additional filtering for secondary structure potential eliminated these positions (designated with an asterisk).

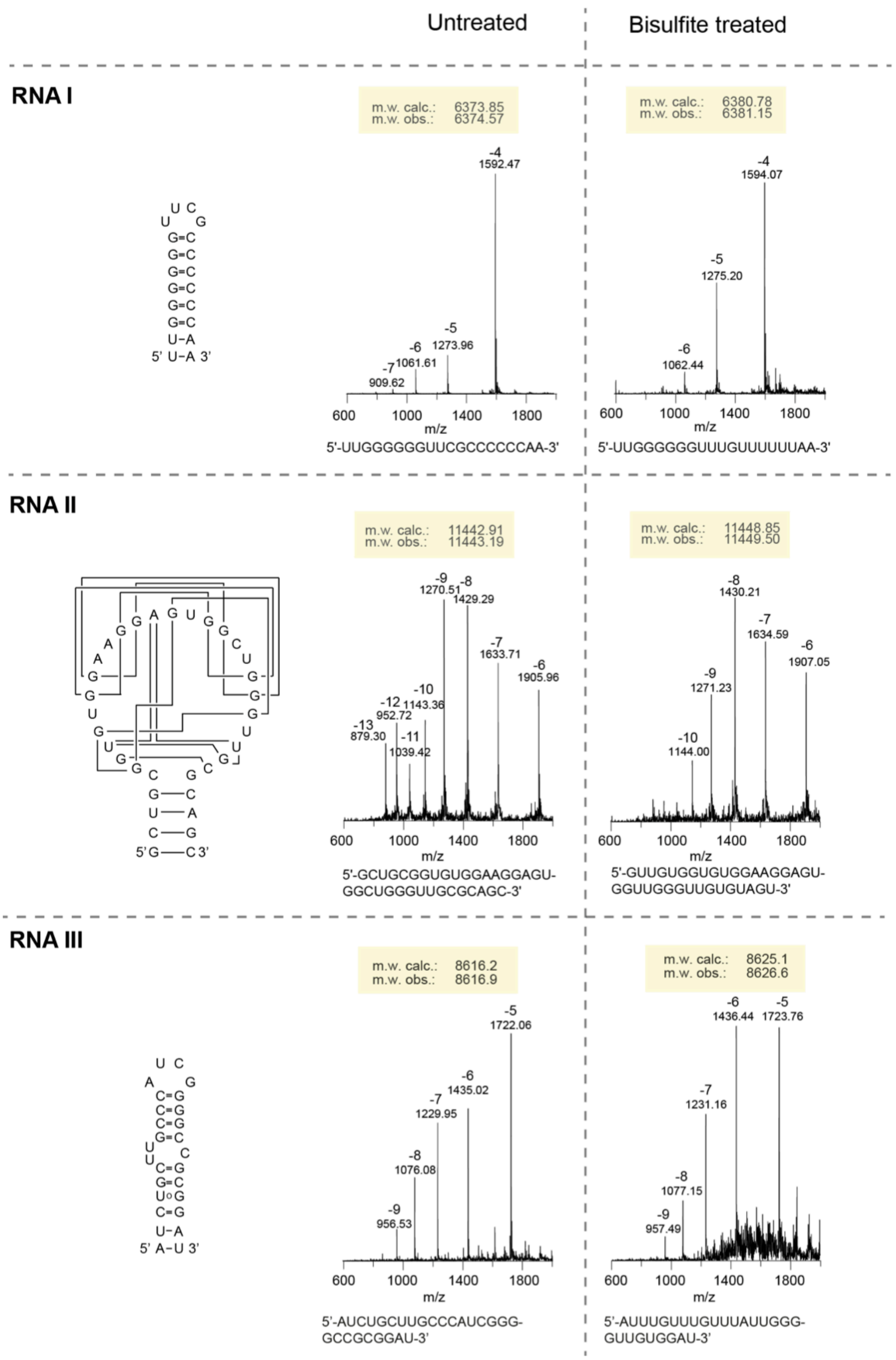

Figure S4. **Mass spectrometry analysis of three unmethylated RNA oligos before and after bisulfite treatment.** The secondary structures of the RNAs are shown at the left, the mass spectra are on the right. The structures for RNAs II and III were determined experimentally [1,2], while the RNA I structure is predicted. The text highlighted in yellow shows the calculated weights versus the observed molecular weights in atomic mass units [amu]. For calculation of the molecular weights after bisulfite treatment, complete conversion of all cytosines to uracil was assumed.

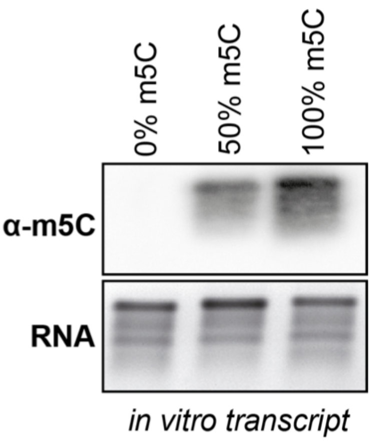

Figure S5. **Immuno-northern blot detection of m5C.** The m5C antibody specifically recognizes m5C in RNA. A 552 nt RNA fragment of *E. coli* 16S rRNA was generated by *in vitro* transcription in the presence of 0, 50 or 100% m5C ribonucleotides, separated on a 1.2% denaturing RNA gel and blotted to a nylon membrane. The membrane was incubated with anti-m5C antibody and detected using ECL (upper panel), the ethidium bromide-stained RNA is shown in the lower panel.

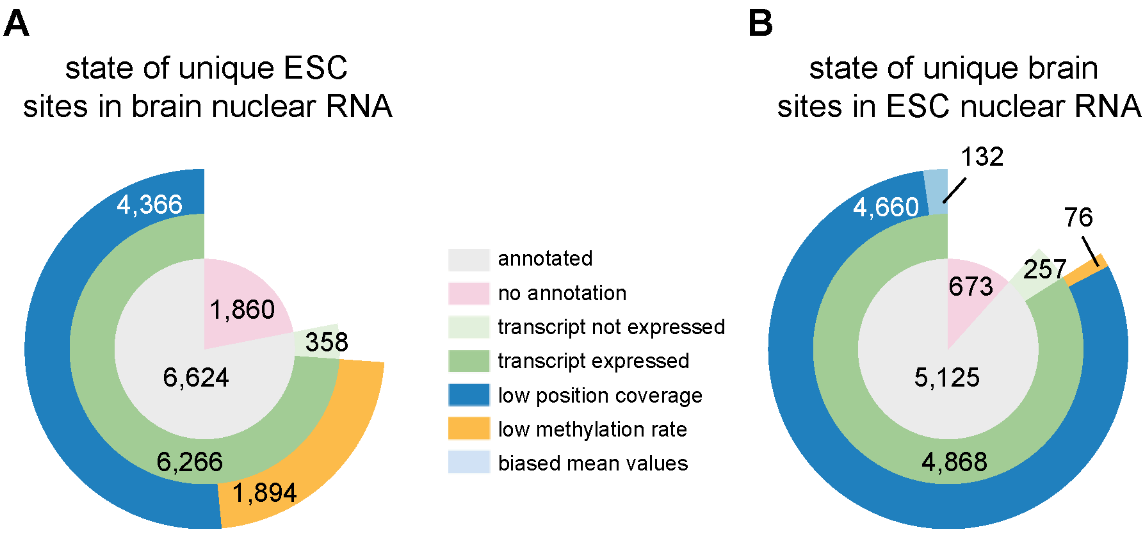

Fig. S6 Analysis of cytosines uniquely methylated in nuclear poly(A)RNA of ESCs or brain. a The expression levels and methylation rates of m5Cs identified as unique to ESCs were analyzed in the brain samples. **b** The expression levels and methylation rates of m5Cs identified as unique to brain were analyzed in the ESC samples. Multi-level pie charts display the numbers of sites on annotated and non-annotated transcripts in the innermost ring, the numbers of sites on transcripts with a mean normalized count of more (dark green) or fewer (light green) than 10 reads in the middle ring, and the numbers of sites with sequence coverage <10 reads (blue) or sequence coverage >10 reads but methylation rate lower than 0.2 (yellow) in the outer ring. Positions in which the mean values for coverage and non-conversion were skewed towards methylation by an individual replicate were classified as “biased mean”.

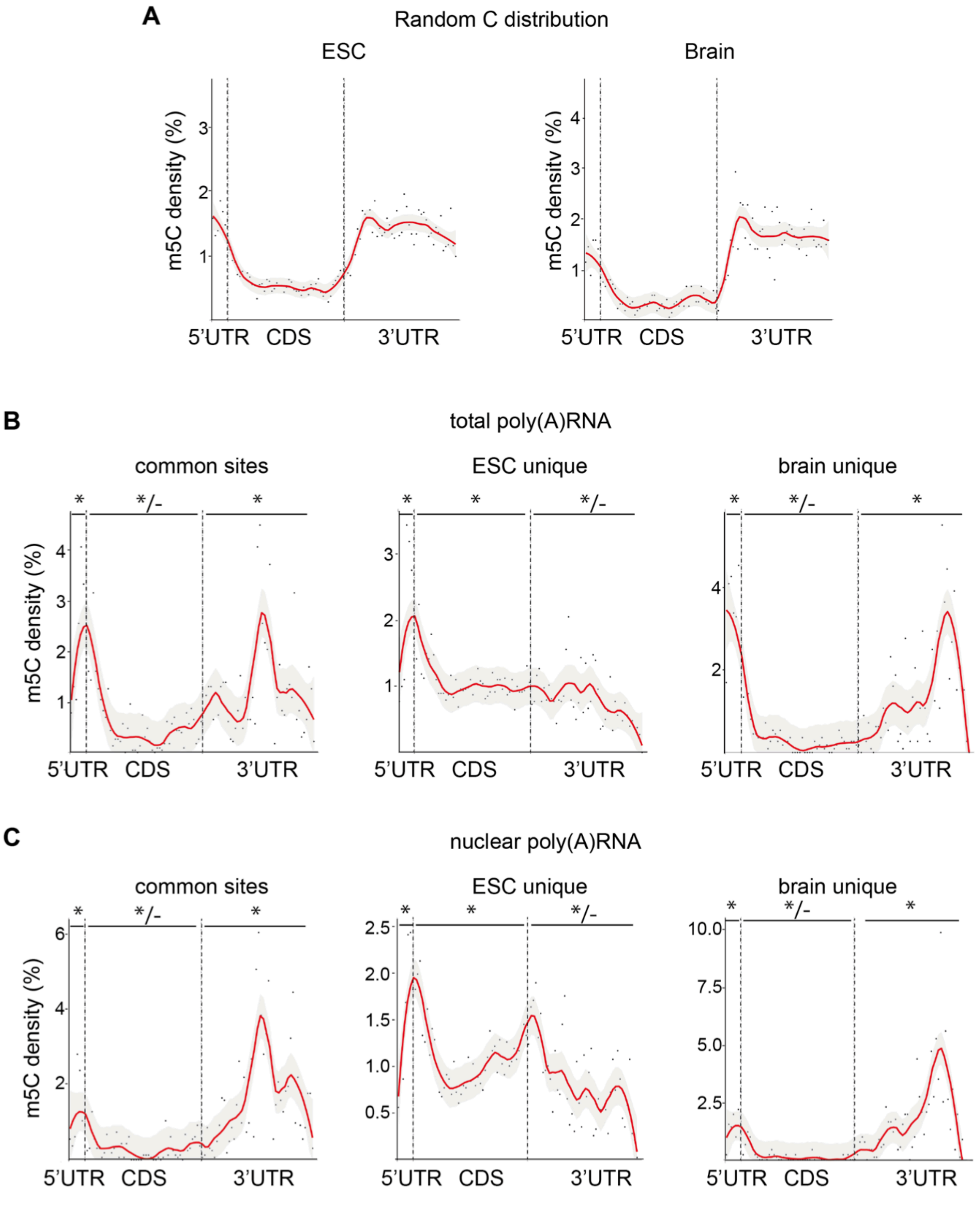

Figure S7. Metagene profiles of m5C distribution in total and nuclear poly(A)RNA from ESCs and brain. a Metagene profiles of Cs randomly sampled from the transcripts that showed methylation. The random C data set was of identical size as the m5C dataset from the different samples. Random C profiles for total poly(A)RNA from ESC and brain are shown, profiles for nuclear poly(A)RNA look very similar and are not shown. **b** Transcripts methylated in total poly(A)RNA of ESCs and brain (*common*) or specifically in ESCs and brain only (*unique*) are shown. **c** Transcripts methylated in

nuclear poly(A)RNA of ESCs and brain (*common*) or specifically in ESCs and brain only (*unique*) are shown. Metagene segments 5'UTR, CDS and 3'UTR are indicated. Enrichment or depletion of m5C in a particular segment was analyzed by Fisher exact and hypergeometric test with a significance threshold of $p<0.05$. Significant enrichment is indicated by *, significant depletion is indicated by */-.

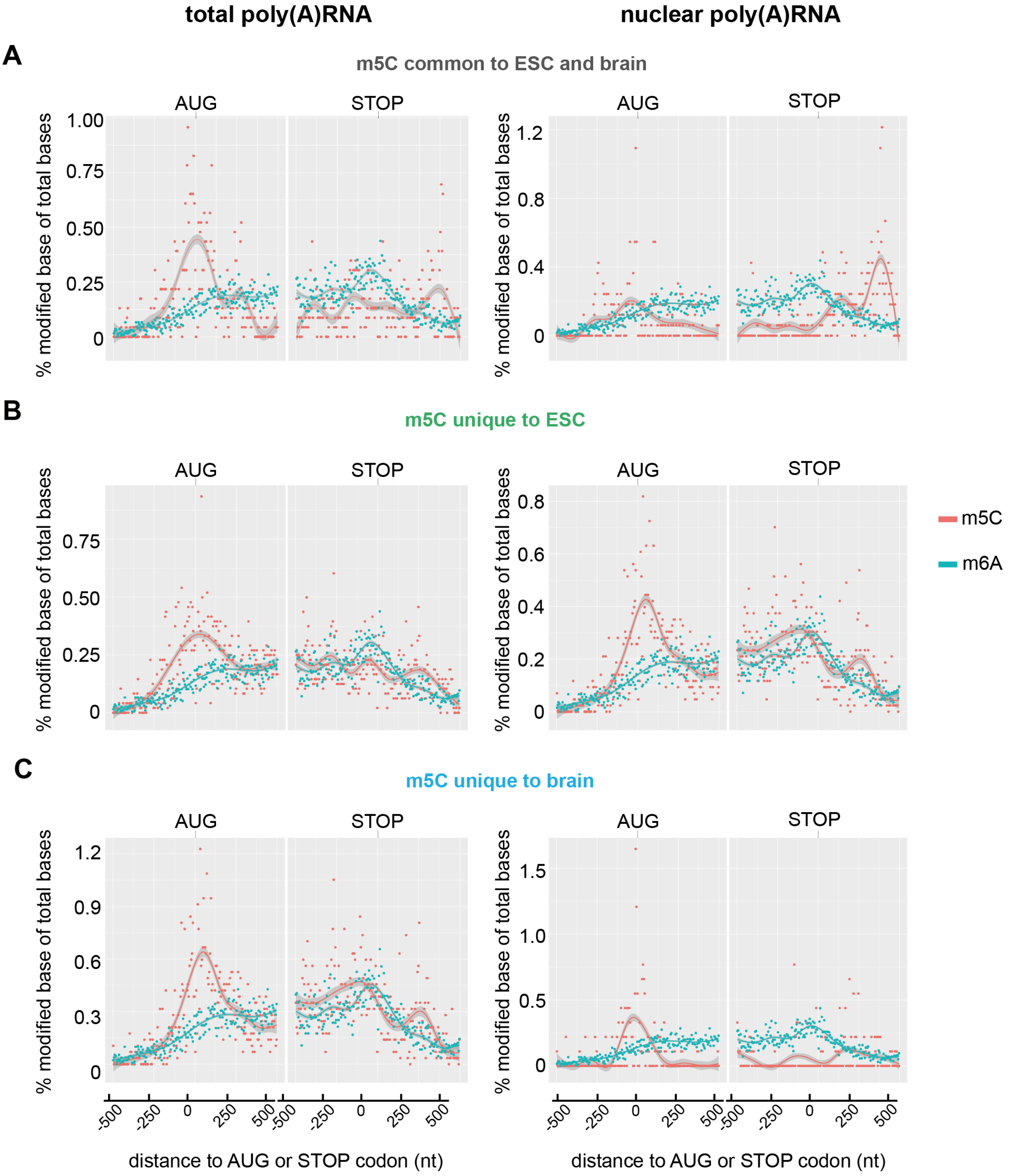

Figure S8. Distribution of m5C and m6A around translational start and stop codons. a The frequency of m5C in total poly(A)RNA (*left*) or nuclear poly(A)RNA (*right*) of ESCs and brain within +/- 500 nt around the translational start (*AUG*) and stop codons is shown. m6A data were retrieved from MeT-db and plotted along the m5C data for comparison. **b** Same as in **a** for m5C positions unique to ESCs. **c** Same as in **a** for m5C positions unique to brain.

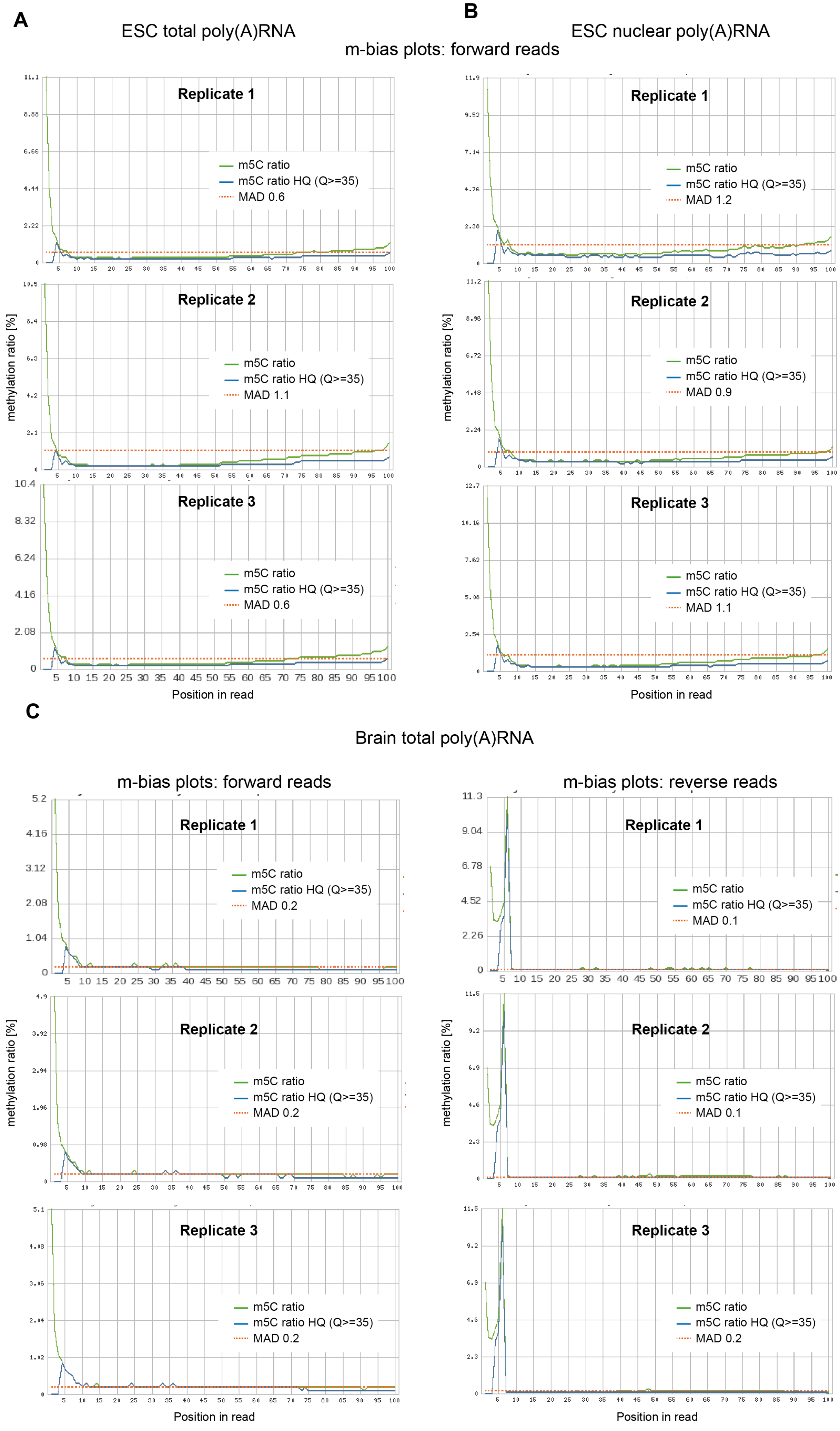
A
ESC total poly(A)RNA
B
ESC nuclear poly(A)RNA
m-bias plots: forward reads
Replicate 1
m5C ratio
m5C ratio HQ (Q>=35)
MAD 0.6
Replicate 2
MAD 1.1
Replicate 3
MAD 0.6
MAD 1.2
MAD 0.9
MAD 1.1
methylation ratio [%]
Position in read
C
Brain total poly(A)RNA
m-bias plots: forward reads
m-bias plots: reverse reads
MAD 0.2
MAD 0.1
MAD 0.2
MAD 0.2
MAD 0.1
MAD 0.2

D

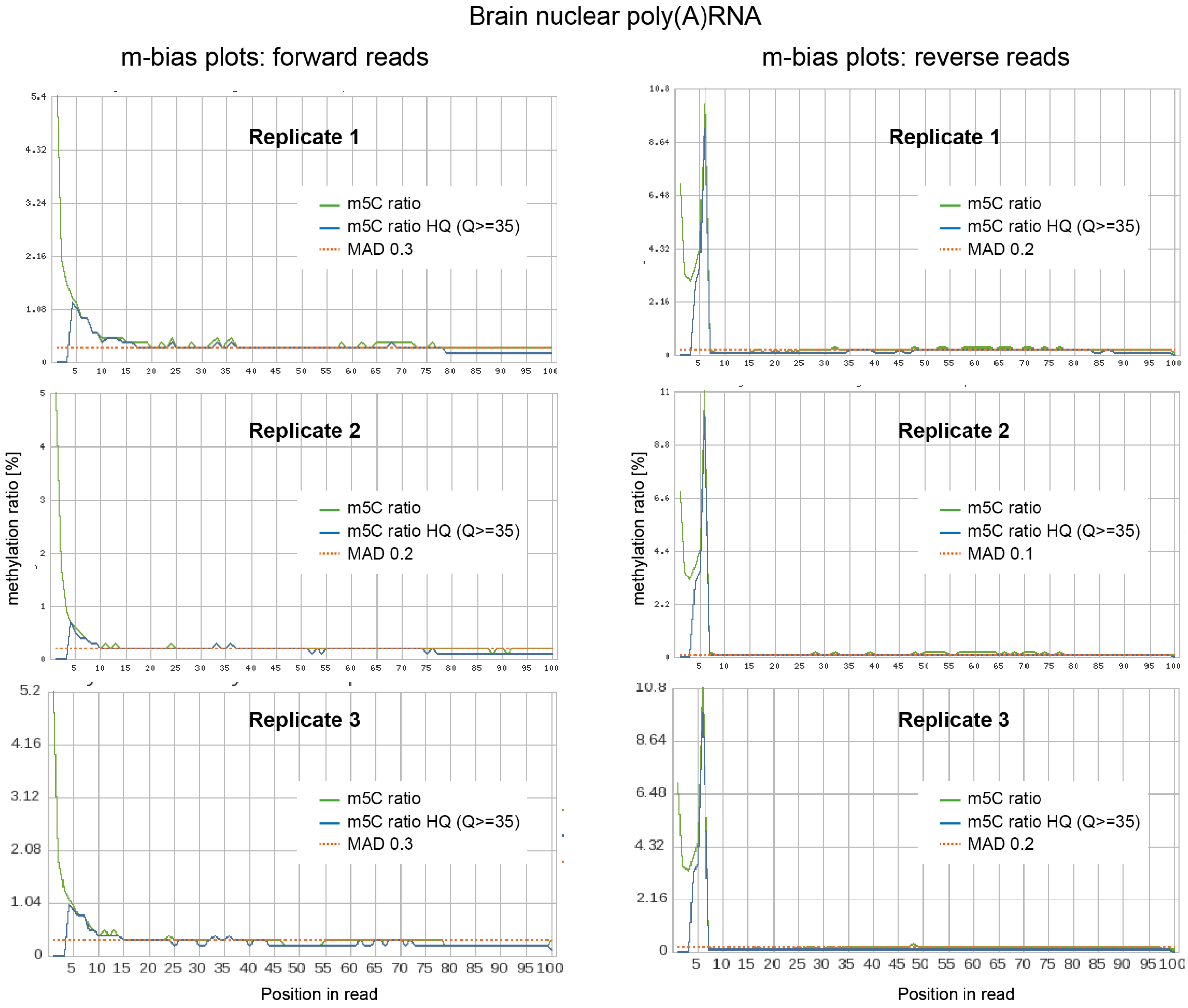

Figure S9. Cytosine 5 methylation bias plots. a, **b** m-bias of single-end reads of ESC total (**a**) and nuclear (**b**) poly(A)RNA samples. **c, d** m-bias of paired-end reads of brain total (**c**) and nuclear (**d**) poly(A)RNA samples. MAD, median plus 2x median absolute deviation.
